# A comparative investigation of the chemical reduction of graphene oxide for electrical engineering applications†

**DOI:** 10.1039/d3nr04521h

**Published:** 2023-10-20

**Authors:** Tomasz Chudziak, Verónica Montes-García, Włodzimierz Czepa, Dawid Pakulski, Andrzej Musiał, Cataldo Valentini, Michał Bielejewski, Michela Carlin, Aurelia Tubaro, Marco Pelin, Paolo Samorì, Artur Ciesielski

**Affiliations:** a Faculty of Chemistry, Adam Mickiewicz University Uniwersytetu Poznańskiego 8 Poznań Poland; b Center for Advanced Technologies, Adam Mickiewicz University Uniwersytetu Poznańskiego 10 Poznań Poland ciesielski@unistra.fr; c University of Strasbourg CNRS ISIS UMR 7006 8 Alleé Gaspard Monge F-67000 Strasbourg France samori@unistra.fr; d Institute of Molecular Physics, Polish Academy of Sciences M. Smoluchowskiego 17 60-179 Poznań Poland; e Department of Life Sciences, University of Trieste Via Fleming 22 34127 Trieste Italy

## Abstract

The presence of oxygen-containing functional groups on the basal plane and at the edges endows graphene oxide (GO) with an insulating nature, which makes it rather unsuitable for electronic applications. Fortunately, the reduction process makes it possible to restore the sp^2^ conjugation. Among various protocols, chemical reduction is appealing because of its compatibility with large-scale production. Nevertheless, despite the vast number of reported chemical protocols, their comparative assessment has not yet been the subject of an in-depth investigation, rendering the establishment of a structure–performance relationship impossible. We report a systematic study on the chemical reduction of GO by exploring different reducing agents (hydrazine hydrate, sodium borohydride, ascorbic acid (AA), and sodium dithionite) and reaction times (2 or 12 hours) in order to boost the performance of chemically reduced GO (CrGO) in electronics and in electrochemical applications. In this work, we provide evidence that the optimal reduction conditions should vary depending on the chosen application, whether it is for electrical or electrochemical purposes. CrGO exhibiting a good electrical conductivity (>1800 S m^−1^) can be obtained by using AA (12 hours of reaction), Na_2_S_2_O_4_ and N_2_H_4_ (independent of the reaction time). Conversely, CrGO displaying a superior electrochemical performance (specific capacitance of 211 F g^−1^, and capacitance retention >99.5% after 2000 cycles) can be obtained by using NaBH_4_ (12 hours of reaction). Finally, the compatibility of the different CrGOs with wearable and flexible electronics is also demonstrated using skin irritation tests. The strategy described represents a significant advancement towards the development of environmentally friendly CrGOs with *ad hoc* properties for advanced applications in electronics and energy storage.

## Abstract 2

Various reducing agents and reaction times are systematically investigated to enhance the performance of chemically reduced GO (CrGO) in electronics and electrochemical applications.

## Graphical abstract

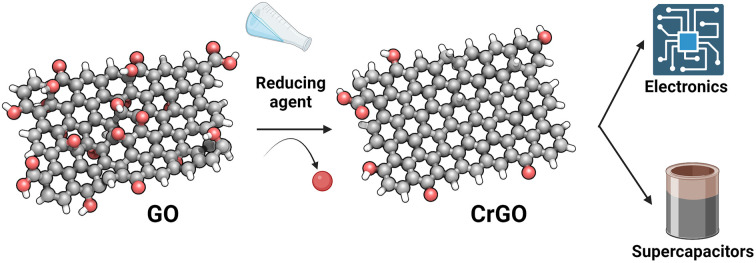

## Introduction

Graphene oxide (GO) is one of the most widely studied two-dimensional materials (2DMs) due to its large-scale production at low cost and easy processing.^1,2^ Oxygen-containing functional groups (OFGs) (hydroxyl, epoxy, carbonyl and carboxyl) present on the basal plane and the edges impart to GOs a unique set of physicochemical properties, such as good dispersibility and colloidal stability in many solvents, including water. Importantly, these OFGs can serve as active sites for chemical modification with multiple molecules, making GO a suitable material for application in the fields of chemical sensing,^3^ energy storage,^4^ water desalination,^5^ drug delivery,^6^ solar cells,^7^ memory devices,^8^ and healthcare^9–11^ to name a few. However, the OFGs present in GO also disrupt the extended sp^2^ network characteristic of graphene, resulting in an insulating material unsuitable for electricity-based applications.^12^ Fortunately, such a limitation can be overcome to a great extent through the removal of OFGs from GO *via* reduction processes, enhancing the degree of conjugation in the carbon network through the formation of sp^2^ species, ultimately boosting the electrical characteristics of the material.^13^ In order to obtain the electroactive form of GO, named reduced graphene oxide (rGO), a variety of thermal (TrGO), chemical (CrGO), electrochemical,^14,15^ sonification,^16^ microwave,^17^ and photo-assisted^18^ methods have been explored,with TrGO and CrGO methodologies being the most extensively employed.^19,20^

On the one hand, thermal reduction represents one of the most attractive reduction methods due to its low environmental impact. However, the high temperatures typically required to accomplish this process (above 1000 °C) are energetically demanding and are incompatible with the use of plastic substrates often desired for flexible electronic applications.^21^ Recently, we have reported a systematic study on the low-temperature annealing of GO by optimizing different annealing conditions, *i.e.*, temperature, time and reduction atmosphere.^4^ We have demonstrated that TrGO can be obtained under air or inert atmosphere at relatively low temperatures (<300 °C) exhibiting low film resistivities (10^−2^–10^−4^ Ωm) combined with unaltered resistance after 2000 bending cycles when supported on plastic substrates. Besides, TrGO electrodes displayed enhanced electrochemical performance, achieving a specific capacitance of 208 F g^−1^ and a capacitance retention >99% after 2000 charge–discharge cycles.^4^

On the other hand, chemical reduction is currently the most efficient approach for reducing GO, approaching the electrical characteristics of graphene (8.5 × 10^4^ S m^−1^ is the highest electrical conductivity reported for CrGO).^22^ Chemical reduction is appealing from the industrial point of view because of its compatibility with large-scale commercial production with low-energy consumption (temperature of reduction is usually below 100 °C). CrGO can be obtained by using a plethora of reducing agents including hydrazine hydrate (N_2_H_4_), dimethylhydrazine, *p*-phenylene diamine, ethylenediamine, hydroxylamine,^23,24^ lithium aluminium hydride, sodium borohydride (NaBH_4_),^25^ sodium bisulfite (NaHSO_3_), l-ascorbic acid (AA),^26–28^ or sodium dithionite (Na_2_S_2_O_4_), among others.

However, a variety of reducing agents and protocols have been reported in the literature (Table S2, ESI†), making it quite difficult to assess and compare these strategies from the perspective of structure–performance relationship. For instance, for applications in electronics, CrGO with the highest electrical conductivity is desirable and therefore the removal of OFGs should be maximized. Unfortunately, some of the employed reducing agents or their oxidized forms may also result in doping or contamination of the CrGO. Although CrGO synthesized with hydrazine hydrate is usually considered to most closely resemble pristine graphene in terms of electronic and structural properties, it has been reported that films of CrGO synthesized using sodium borohydride have significantly lower sheet resistance. This result can be explained by CrGO contamination with nitrogen and pyrazole formation, where the nitrogen atoms behave as electron donors and supply p-type holes.^25,29,30^ Ascorbic acid, which can be regarded as the most studied ‘green’ reducing agent to obtain CrGO, also suffers from the same contamination problem.^26,28,31–33^ AA is oxidized first to dehydroascorbic acid (DHA) and then to oxalic and guluronic acids, both of which can supramolecularly interact with the unreacted carboxylic groups of pristine GO.^34–36^

In contrast to electrical applications, the scenario is not as straightforward when it comes to electrochemical energy storage applications.^37,38^ As previously reported, OFGs contribute towards enhancing the electrochemical performance of pseudocapacitive rGO electrodes and their removal is always detrimental to the device performance. For instance, ultrahigh-level oxygen-functionalized GO (UHFGO) has shown an impressive capacitance of 285 F g^−1^ in a gel electrolyte.^39^ Alternative strategies such as heteroatom doping have also been widely employed to boost the electrochemical performance of pristine GO.^40,41^ Nevertheless, the electrochemical performance of rGO electrodes is superior to that of pristine GO electrodes because the removal of OFGs is accompanied by other physicochemical and structural changes in rGO, such as an increase in conductivity, surface area and pore size. Therefore, the best reducing conditions for applications in electronics or in electrochemical energy storage may differ. To the best of our knowledge, there is no systematic study that compares the physicochemical and structural properties of CrGO with its performance in electronics or in electrochemical energy storage by using different reducing agents and reducing times. Furthermore, one of the major criticisms that chemical reduction has received is the use of toxic reducing agents. However, there has been limited discussion regarding the toxicity of the resulting CrGO.

To address this significant knowledge gap, we investigated the fine-tuning of the reduction degree of GO by varying the reduction conditions, specifically the choice of the reducing agent and reaction time (2 or 12 hours). Regarding reducing agents, we focused our attention on four of the most commonly employed ones, namely, hydrazine hydrate, sodium borohydride, ascorbic acid, and a sulphur-containing compound such as sodium dithionite.^42,43^ Our study was aimed at systematically investigating how these factors influenced the toxicity and the physicochemical and structural properties of CrGO, ultimately boosting their performance for electronic devices or energy storage applications.

## Results and discussion

The chemical reduction of GO was firstly followed by Fourier-transform infrared (FTIR) spectroscopy (Fig. S1, ESI†). The black curve reveals the characteristic vibration bands of GO: 2500–3500 cm^−1^ (OH, stretching vibration), 1722 cm^−1^ (C=O, stretching vibration), 1620 cm^−1^ (aromatic C=C, stretching vibration), 1400 cm^−1^ (C–OH, bending vibration), 1220 cm^−1^ and 1046 cm^−1^ (breathing vibrations) and ∼1000 cm^−1^ (stretching vibrations from the epoxy, ether or peroxide groups).^28,34,44^ After 2 hours of chemical reduction, the vibrations related to the different OFGs were significantly reduced in rGO(N_2_H_4_) and rGO(Na_2_S_2_O_4_). However, to observe the same reduction degree in rGO(AA) or rGO(NaBH_4_), 12 hours of reduction was needed. Based on the ratio of the C=C stretching vibration to any OFG vibration, FTIR analysis offered the first insight into the reduction strength of the four reducing agents explored, with N_2_H_4_ and Na_2_S_2_O_4_ as the strongest reducing agents, then AA and finally NaBH_4_ as the mildest reducing agent.

The degree of GO reduction is usually expressed by the C/O ratio obtained from X-ray photoelectron spectroscopy (XPS) analysis. However, as XPS is a surface-sensitive technique (*i.e.*, the penetration depth of the XPS beam ranges only between 1 and 10 nm), we firstly performed elemental analyses (E.A.) of chemically reduced GO (Table S3, ESI†). As can be seen in Table S3,† the C/O ratio ranges from 0.99 for pristine GO to 7.20 for rGO(N_2_H_4_)_12 h. According to the C/O ratio, the strength of the reducing agents varies as follows: Na_2_S_2_O_4_ ≈ N_2_H_4_ > AA > NaBH_4_, in agreement with FTIR analyses. Interestingly, the nitrogen content is slightly higher in the samples reduced with N_2_H_4_, indicating a possible contamination from the reaction of GO with N_2_H_4_ (*e.g.*, pyrazole formation).

**Fig. 1:**
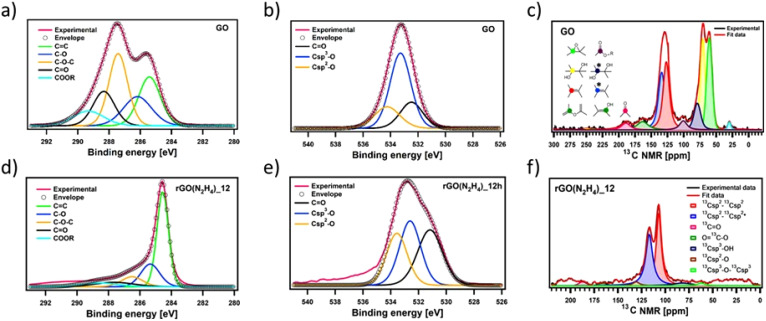
Fitted XPS (a and d) C 1s and (b and e) O 1s spectra of (a and b) GO and (d and e) CrGO with hydrazine for 12 hours of reaction time and their corresponding chemical groups; (c and f) fitted ssNMR spectra of (c) GO and (f) CrGO with N_2_H_4_ for 12 hours of reaction time and their corresponding chemical groups. The stars refer to the chemical groups close to defects.

**Fig. 2:**
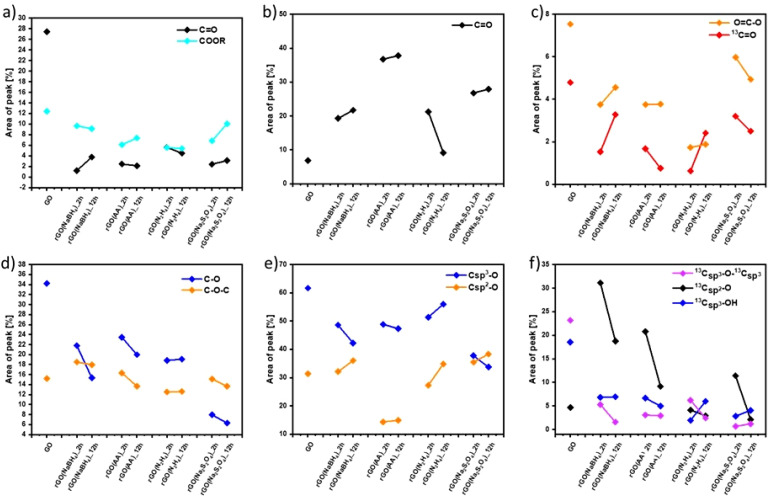
Comparison of the relative contribution of (a and d) C 1s XPS, (b and e) O 1s XPS and (c and f) ssNMR spectral components as a function of the reducing agents and reaction time estimated by dividing the area under each component by the whole peak area.

To gain insights into the chemical composition of CrGO, X-ray photoelectron spectroscopy (XPS) and solid-state NMR magic angle spinning (ssNMR-MAS) analyses were then performed on the GO and CrGO powders (Fig. 1–2 and Fig. S2–10, ESI†). From the XPS survey spectra (Fig. S2 and 3, ESI†), the C/O ratio was estimated (Table S4, ESI†), ranging from 0.86 for pristine GO up to 11.21 for rGO(Na_2_S_2_O_4_)_12 h. In full agreement with FTIR and E.A., the strength of the reducing agents followed the same trend. Likewise, the nitrogen element was also present in the rGO(N_2_H_4_) samples, and its amount was proportional to the reaction time (2.70% after 12 hours of reaction). Traces of sodium element (∼1%) were found in the rGO(NaBH_4_) samples, but were not sufficient to be detected in elemental analysis. No contamination of sodium or sulfur was observed in the rGO(Na_2_S_2_O_4_) samples. Regarding the reaction time, a negligible difference (*i.e.*, max. 5% of increase) was obtained between 2 and 12 hours of reaction for all the reducing agents. Although FTIR, E.A. and XPS survey spectra showed the same tendency in the strength of the reducing agents, a deeper analysis was needed to unveil the influence of the reaction time. To cast light onto the chemistry of the reduction process by each reducing agent, the high resolution C 1s and O 1s XPS and ssNMR-MAS spectra were deconvoluted as previously reported by us.^4^ The high resolution C 1s spectra were fitted using 5 Gaussian–Lorentzian curves for the 5 chemical environments: 284.5 eV C–C (C_sp^2^_–C_sp^2^_), 285.15 eV C–O (including C_sp^2^_–O–C_sp^2^_, C_sp^3^_–OH and C_sp^2^_–OH), 286.5 eV C–O–C (C_sp^3^_–O–C_sp^3^_), 287.40 eV C=O, and 288.50 eV COOR (including COOH and lactone) (Fig. 1(a), (d) and Fig. S4, ESI†).^4^ Likewise, the high resolution O 1s spectra were fitted with 3 Gaussian–Lorentzian curves: 531.08 eV C=O, 532.03 eV C_sp^3^_–O (including C_sp^3^_–O–C_sp^3^_, and C_sp^3^_–OH), and 533.43 eV C_sp^2^_–O (including C_sp^2^_–O–C_sp^2^_ and C_sp^2^_–OH) (Fig. 1(b), (e) and Fig. S5, ESI†).^4^ To complete the analysis, the ssNMR-MAS spectra were deconvoluted in eight curves: 60.4 ppm (^13^C_sp^3^_–O–^13^C_sp^3^_), 70.6 ppm (^13^C_sp^3^_–OH), 78.9 ppm (^13^C_sp^3^_–OH, close to defects), 100.2 ppm (^13^C–OOR), 126.7 ppm (^13^C_sp^2^_–^13^C_sp^2^_), 134.7 ppm (^13^C_sp^2^_–^13^C_sp^2^_ close to defects), 162.4 ppm (C_sp^2^_–O (including C_sp^2^_–O–C_sp^2^_ and C_sp^2^_–OH)) and 187.9 ppm (^13^C=O) (Fig. 1(c), (f) and Fig. S8, ESI†). Fig. 1 shows the deconvolution of the C 1s and O 1s XPS and ssNMR-MAS spectra for GO (Fig. 1(a–c)) and as representative CrGO, we show analogous results using rGO(N_2_H_4_)_12 h (Fig. 1(d–f)). The results for all the reducing agents and reaction times can be seen in the ESI (Fig. S4–5 and S8†).

Fig. S7 and S9 (ESI†) show the evolution of the area of each component in XPS (Fig. S7, ESI†) and ssNMR spectra (Fig. S9, ESI†) as a function of the reducing agents and reaction time. However, for a straightforward analysis, we divided the components into three groups, (i) C=C (Fig. S10, ESI†), (ii) C=O (Fig. 2(a–c)) and (iii) C–O (Fig. 2(d–f)). As expected, the area of the C=C (Fig. S10(a), ESI†) and C_sp^2^_–C_sp^2^_ (Fig. S10(b), ESI†) peaks increased in all the cases as the reduction process restored the π-conjugation of the carbon sheets. While the largest C=C and C_sp^2^_–C_sp^2^_ peak areas were obtained for rGO(Na_2_S_2_O_4_)_2 h, the smallest C=C and C_sp^2^_–C_sp^2^_ peak areas were observed for rGO(NaBH_4_)_2 h. The reaction time only played a major role when NaBH_4_ was used as a reducing agent. The area of C_sp^2^_–C_sp^2^_ close to the defects (C_sp^2^_–C_sp^2^_*) increased with the reduction process. Interestingly, we observed a decrease of the C_sp^2^_–C_sp^2^_* peak area after 12 hours of reaction when NaBH_4_ and N_2_H_4_ were used. This can be directly correlated with the contamination with the sodium and nitrogen elements, respectively, which reacted with the defects present in the GO sheets. To demonstrate the reactivity between GO and N_2_H_4_, the high-resolution N 1s XPS spectra were obtained (Fig. S6, ESI†) at 2 and 12 hours of reaction. Fig. S6 (ESI†) shows the formation of a pyridine ring, C–N–C and a pyrrole or diazine ring (N–C=O).^45,46^ Among the different OFGs present in GO, carbonyl groups, either in the form of C=O or COOR, were present in the lowest percentage. Fig. 2(a–c) shows that N_2_H_4_ is the most efficient reducing agent for the COOR groups, whereas all reducing agents yielded similar amounts of C=O moieties. The evolution of the area corresponding to the four different C–O species present in GO can be seen in Fig. 2(d and f). The peak area of C–O–C or ^13^C_sp^3^_–O–^13^C_sp^3^_ (Fig. 2(d) and (f), respectively) decreased after chemical reduction and no major differences were found between all the reducing agents or the reaction times. The same trend was observed for C_sp^3^_–O peaks (which include both the C_sp^3^_–O–C_sp^3^_ and C_sp^3^_–OH peaks) (Fig. 2(e)) but Na_2_S_2_O_4_ showed a higher decrease (nearly 30% compared to GO). Finally, the most interesting findings were revealed by the C_sp^2^_–O species (which included both C_sp^2^_–O–C_sp^2^_ and C_sp^2^_–OH). As we previously demonstrated, during the reduction process there was a structural reorganization of C_sp^3^_–O to C_sp^2^_–O and therefore we observed an increase in the peak area of the C_sp^2^_–O species. However, when the reduction continued, the OFGs were eliminated, leading to a decrease in the peak area of the C_sp^2^_–O species. This phenomenon can be clearly observed by ssNMR (Fig. 2(f)). For the mildest reducing agent rGO(NaBH_4_)_2 h, there was a sharp increase of the C_sp^2^_–O peak area compared to that of pristine GO, which then decreased when the reduction was performed for 12 hours. The same trend was observed when rGO(AA)_2 h, the following reducing agent by strength, was used. However, the peak area increase was lower than that in rGO(NaBH_4_)_2 h and when the reduction was performed for 12 hours the peak area of C_sp^2^_–O was comparable to that of pristine GO. Interestingly, when N_2_H_4_ was used, no increase in the peak area of C_sp^2^_–O was observed, independent of the reaction time. Finally, rGO(Na_2_S_2_O_4_)_2 h followed the same trend as rGO(AA) but with a lower peak area increase. Therefore, ssNMR unequivocally proves that the strength of the reducing agents varies as follows: Na_2_S_2_O_4_ ≈ N_2_H_4_ > AA > NaBH_4_. Besides, the reaction time plays a role for three of the reducing agents, Na_2_S_2_O_4_, AA and NaBH_4_.

Raman spectroscopy confirmed not only the chemical composition of CrGO but also that the different Raman features (*e.g.*, band position, intensity ratio and width) were related to structural parameters such as crystallinity, reduction degree of GO and oxygen content.^47^ The Raman spectra of GO and CrGO were deconvoluted by using five Lorentzian curves, which consist of the first-order Raman modes, namely: D, D′′, D′, D* and G (Fig. S11 and 12, ESI†). While the D band (∼1350 cm^−1^) is associated with the breathing modes of photons of A_1g_ symmetry, the G band (∼1585 cm^−1^) is related to the first-order scattering of E_2g_ phonons of the sp^2^ carbon structure.^48^ Commonly, the relative intensity of the D band with respect to the G band (*I*_D_/*I*_G_ ratio) is an insightful parameter to estimate the degree of defects in GO derivatives and it has been correlated with the inverse of the crystallite size on basal planes (1/*L*_a_) by Tuinstra and Koenig.^49^ Fig. S13(a) (ESI†) reveals that the *I*_D_/*I*_G_ ratio increases for all the reducing agents, indicating the restoration of sp^2^ conjugation due to the removal of the oxygen functional groups from GO.^50,51^ The increase in the *I*_D_/*I*_G_ ratio is proportional to the strength of the reducing agent, in full agreement with previous characterization results. In addition, the *I*_D_/*I*_G_ ratio decreases after 12 hours of reaction when AA and N_2_H_4_ are employed, which is related to undesired contamination. The additional bands (D′′, D* and D′) arise from the defects present in the graphitic structure of the carbon material.^48,52–54^ The *I*_D′′_/*I*_G_, *I*_D*_/*I*_G_, and *I*_D′_/*I*_G_ ratios are shown in Fig. S13(b), (c) and (d) (ESI†), respectively. Ideally, all these ratios should decrease with the reduction degree but as we can see, the *I*_D′_/*I*_G_ and *I*_D*_/*I*_G_ ratios increased slightly in all the cases; therefore, it can be concluded that the chemical reduction process creates defects in the graphitic structure of the carbon material. In contrast, the *I*_D′′_/*I*_G_ ratio decreases in all the cases and the decrease is proportional to the strength of the reducing agent.

To unravel the number of defects present in the pristine GO and CrGO we followed the protocol reported by Cançado *et al.*^51^ First, we calculated the average defect distance (*L*_D_) by using eqn (2) (see the Materials and characterization section in the ESI† for details). As shown in Fig. S14(a),† the *L*_D_ of pristine GO amounts to 9.64 ± 1.11, which upon reduction only decreases slightly to 8.27 ± 0.95 for the strongest reducing conditions (rGO(Na_2_S_2_O_4_)_12 h). As *L*_D_ ≈ 10, we estimated the number of defects in each case by using eqn (3) (see the Materials and characterization section in the ESI† for details). Fig. S14(b)† shows that the number of defects in GO and CrGO is between 7.8 × 10^10^ and 10.6 × 10^10^.

**Fig. 3:**
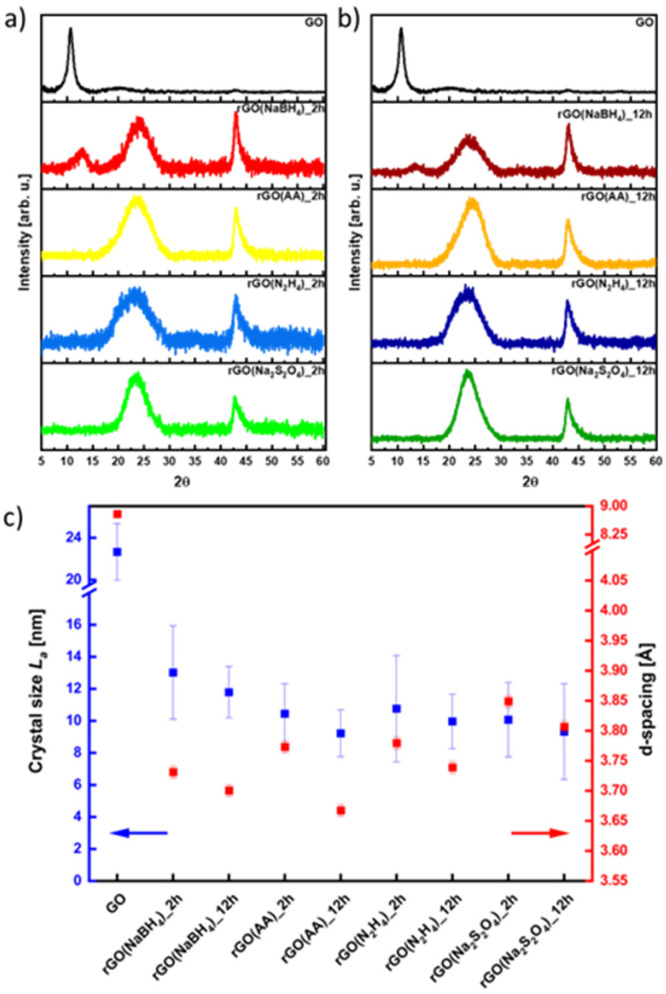
XRD patterns of rGO reduced for (a) 2 hours and (b) 12 hours, and (c) dependence on crystal size (*L*_a_) and *d* -spacing in correlation with the reducing agent.

The effect of the different reducing agents on the crystallinity of CrGO was investigated using powder X ray diffraction (PXRD) (Fig. 3). The pristine GO diffraction pattern displays one characteristic peak at 2*θ* = ∼10° (peak I) with a Full-Width at Half Maximum (FWHM) of 0.81 related to the (002) family of planes (Fig. 3). After the chemical reduction, the CrGO exhibits one characteristic peak at 2*θ* = ∼25° (peak II), with a larger FWHM of 4.4–5.81°, related to the smaller crystallite sizes and a second at about 42.8° related to the (100) family of planes. Fig. 3 shows that after two hours of reaction peak I completely disappears when Na_2_S_2_O_4_, N_2_H_4_ and AA reducing agents are used. However, peak I is still present even after 12 hours of reaction when NaBH_4_ is used, which is in agreement with the results obtained by Shin *et al.*^25^ From the scattering angle (2*θ*) of each peak we can quantify the *d*-spacing, average crystalline size (*L*_a_), crystalline thickness (*L*_c_), and graphene layer number (*n*) for GO and all CrGO using the Debye–Scherrer equation (Fig. 3(c) and Table S5, ESI†). The *d*-spacing of pristine GO amounts to 8.79 Å and after its chemical reduction, it decreased to ∼3.76 Å.^55,56^ The considerable shrinkage of the interlayer distance is connected to the partial removal of the OFGs from the GO sheets. The *d*-spacing slightly decreases with the reaction time but no major differences are found between the reducing agents employed. The crystal thickness (*L*_c_) considerably decreases from 97.32 Å of pristine GO to ∼15.5 Å after its chemical reduction. Interestingly, no major differences are found between the reducing agents and reaction time for both *L*_c_ and *L*_a_ (Fig. 3(c) and Table S5, ESI†). Remarkably, the theoretical number of layers (*n*_c_) reveals that few layer-thick CrGO sheets (3–5 layers) can be produced even with mild reducing agents like AA. Therefore, we can conclude that the choice of the reducing agent and reaction time have no strong influence on the crystallinity of the resulting CrGO.

Scanning electron microscopy (SEM) images (Fig. S15 and 16, ESI†) reveal the absence of morphological changes upon the chemical reduction of GO for all the reducing agents and reaction times employed. Thermogravimetric analysis (TGA) was then performed to evaluate the thermal stability of CrGO (Fig. S17, ESI†). As shown in Fig. S17 (ESI†), the thermal stability of GO increased upon chemical reduction and the Td_10_ (thermal decomposition of 10% weight) increased from 70 °C (pristine GO) to 430 °C in the case of Na_2_S_2_O_4_ and N_2_H_4_ and to 450 °C in the case of AA and NaBH_4_. Two main conclusions can be drawn from the TGA analysis: in agreement with the previous characterization results, no major differences were found between the two reaction times studied and the thermal stability of CrGO was the highest for the samples with the highest C/O ratio and therefore characterized by a greater C_sp^2^_ content.

The specific surface area and average pore size of CrGO were evaluated by recording N_2_ adsorption–desorption isotherms at 77 K (Fig. S18–21, ESI†). The adsorption isotherms of the CrGO exhibited type-I sorption isotherms, with steep rises appearing at low relative pressure and type-IV sorption features with adsorption/desorption hysteresis at higher pressure. The calculated Brunauer–Emmett–Teller (BET) surface area of CrGO revealed significant differences between the reducing agents used (Fig. S22(a), ESI†). In all the cases, the surface area increased with the reaction time (between 25 and 97% increase). Interestingly, the two reducing agents that produced CrGO with the highest C/O ratio (Na_2_S_2_O_4_ and N_2_H_4_), exhibited the lowest surface areas (140.91 and 96.41 m^2^ g^−1^, respectively, after 2 hours of reaction). In contrast, when the mildest reducing agents AA and NaBH_4_ were used, CrGO exhibited the highest surface areas (394.90 and 555.63 m^2^ g^−1^, respectively, after 12 hours of reaction) compared to pristine GO (12.61 m^2^ g^−1^). The same trends were observed for the average pore size of CrGO (Fig. S22(b), ESI†), ranging from 1.40 nm when Na_2_S_2_O_4_ was used for 2 hours of reaction time, to 13.70 nm when AA was used for 12 hours of reaction time.

To gain a greater insight into the electrical performance of CrGO, thin film conductivity measurements were performed. Pellets of different materials were prepared (see the ESI†) and the film resistivity was measured with a four-point probe (FPP) (Fig. S23, ESI†). Due to its insulating character, the film resistivity of the pristine GO material was above our instrument's detection limit. The film conductivity of CrGO, ranging from 2.7 × 10^1^ S m^−1^ (rGO(NaBH_4_)_2 h) to 4.3 × 10^3^ S m^−1^ (rGO(Na_2_S_2_O_4_)_2 h), gradually increased with the strength of the employed reducing agent, following a trend in full agreement with the previous characterization results. Therefore, for electrical applications, rGO(AA)_12 h, rGO(Na_2_S_2_O_4_) and rGO(N_2_H_4_) represent the best choices, as their performance is comparable.

To explore the compatibility of our reduction process with substrates employed in flexible electronics, the reduction of a film GO deposited on polyethylene terephthalate (PET) is performed using the four reducing agents. As can be seen in Fig. S24 (ESI†), only the CrGO films reduced with AA and Na_2_S_2_O_4_ are stable and homogeneous. Then, the mechanical stability of the films was tested by performing 2000 bending cycles (Fig. S25, ESI†). The resistance of the film was constant for the 2000 bending cycles performed with subtle variations below 1%.

### Electrochemical characterization

**Fig. 4:**
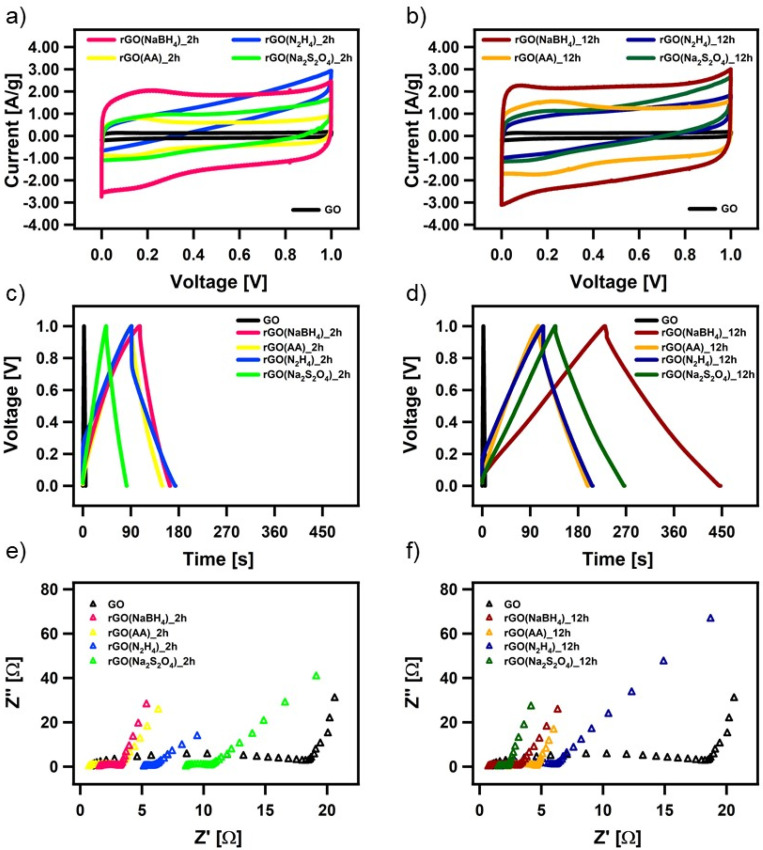
Electrochemical characterization of chemically reduced GO. (a and b) CV curves at a scan rate of 5 mV s^−1^, (c and d) GCD profiles at a current density of 1 A g^−1^ and (e and f) magnification of the high-frequency region of the Nyquist plots.

The electrochemical performance of all the CrGO samples was evaluated in a symmetrical two-electrode cell using cyclic voltammetry (CV), galvanostatic charge/discharge (GCD) and electrochemical impedance spectroscopy (EIS). Fig. 4(a) and (d) show the CV profiles at a 2 mV s^−1^ scan rate of GO reduced with the different reducing agents at (a) 2 and (d) 12 hours of reaction. As we previously demonstrated, rGO is a pseudocapacitor material that agrees with the obtained quasi-rectangular shaped CVs.^4,57,58^ The electrochemical performance can be inferred from the area of the CV plot, providing an initial indication of the most promising samples. In particular, rGO(NaBH_4_), at both 2 and 12 hours of reaction, displays the CVs with the largest area. Fig. S26 (ESI†) shows the CV profiles at different scan rates of CrGO with different reducing agents at different reaction times. The symmetrical capacitive behavior was maintained up to an ultrafast scan rate of 2000 mV s^−1^, implying a quick charge propagation within the electrode material.

The pseudocapacitive behavior of CrGO is confirmed by galvanostatic charge/discharge curves, as shown in Fig. 4(b), (e) and Fig. S27 (ESI†). The voltage–time curve exhibits a quasi-linear shape and similar trends to those obtained by CV analysis. The specific capacitances of CrGO were calculated from GCD profiles at different current densities, as shown in Fig. S28 (ESI†) (see the Materials and characterization section, Experimental details subsection for calculation details). The maximum specific capacitance amounts to 211 F g^−1^ for rGO(NaBH_4_)_12 h. From these values two main conclusions can be drawn: (i) the reaction time plays a major role in the obtained specific capacitance of CrGO for the four reducing agents and (ii) the obtained specific capacitances of CrGO are not directly proportional to the strength of the reducing agents employed.

Surface area, porosity, electrical and ionic conductivity and electrochemical activity have been identified as the key properties that strongly influence the electrochemical performance of materials employed as energy storage systems (ESS). The increase in surface area is directly proportional to the electrochemical performance, as a large surface area offers access to abundant active sites for electrochemical reactions or electrostatic interactions.^59^ As shown in Fig. S29(a) (ESI†), surface area and capacitance follow nearly the same trend for the different reducing agents and reaction times. High porosity is crucial in faradaic charge transfer processes in pseudocapacitive-type materials and determines the access of the charge transfer active sites and facilitates ion conduction through the electrode material.^60^ As in the case of surface area, Fig. S29(b) (ESI†) shows that pore size and capacitance follow the same trend. Higher electrical conductivity facilitates electron migration from electrode materials to current collectors, enabling better rate performance. Fig. S29(c) (ESI†) shows that above a certain threshold (conductivity > 20 S m^−1^) the increase in electrical conductivity is not translated into an increase in capacitance. Ionic conductivity is a critical factor affecting the electrochemical performance of double-layer capacitors and pseudocapacitors. Improved ionic conductivity helps instantaneous polarization across the electrode surface, which permits greater access of the electrolyte ions within a short time. The ionic conductivity of GO and CrGO-based electrodes is obtained using electrochemical impedance spectroscopy (EIS) (see Table S6, ESI†). Fig. S29(d) (ESI†) shows that, except for GO, the ionic conductivity and capacitance follow the same trend. The presence of electrochemically active groups (*e.g.*, OFGs) is advantageous, as they can provide a large additional pseudocapacitance. During the reduction process, OFGs are eliminated and therefore this is detrimental to the electrochemical performance. Fig. S29(e) (ESI†) shows that the two reducing agents that produce CrGO with lower oxygen content (*i.e.*, N_2_H_4_ and Na_2_S_2_O_4_) also show the lowest capacitance values. In contrast, AA and NaBH_4_ produce CrGO with higher oxygen content, but only after 12 hours is the specific capacitance boosted. Pristine GO, despite having the highest amount of OFG, exhibits the lowest capacitance due to its small surface area and poor conductivity.

All CrGO samples show a good rate capability, with a decrease of less than 30% in capacitance at higher current densities. For instance, the capacitance of rGO(NaBH_4_)_12 h is as high as 169 F g^−1^ at a high current density of 20 A g^−1^, implying a quick charge propagation. Another important factor affecting the electrochemical performance of supercapacitor devices is the Ohmic drop (IR), which is caused by internal resistance (including the electrolyte resistance, the resistance of the electrode active-material, and the contact resistance between the active materials and the current collector).^61,62^ In the case of the rGO(NaBH_4_), rGO(AA), and rGO(Na_2_S_2_O_4_) electrodes, we can observe a very small IR drop (∼2–3%) while the rGO(N_2_H_4_) electrodes exhibit a significantly larger IR drop (∼20%), which indicates a non-effective discharging process.

Moreover, the EIS data are evaluated by examining the Nyquist plots (shown in Fig. 4(c) and (f)). In an ideal double-layer capacitor, the Nyquist plot should appear as a vertical line running parallel to the imaginary axis. However, the presence of a semicircle at high frequencies indicates the existence of various OFGs on the GO surface, giving rise to a pseudocapacitive behavior. The experimental results are well-fitted with the indicated circuit (Fig. S30, ESI†) and the fitting parameters can be seen in Table S6 (ESI†). The low *R*_ct_ values of CrGO are consistent with the fact that at a current density, as high as 20 A g^−1^, a high value of capacitance is still obtained, indicating a good rate capability of the CrGO samples. Besides, among the different CrGO samples, rGO(N_2_H_4_)_12 h shows the highest *R*_ct_, which is in agreement with the observed largest IR drop.

The long-term stability of the prepared CrGO electrodes was then investigated using galvanostatic charge–discharge cycles at a current density of 1 A g^−1^ (Fig. S31, ESI†). The samples exhibited excellent long-term stability Independent of the reducing agent and reaction time. For instance, CrGO at 12 hours of reaction time exhibited retention rates of 99.5%, 97.5%, 97% and 95% of the initial capacitance for rGO(NaBH_4_), rGO(AA), rGO(Na_2_S_2_O_4_), rGO(N_2_H_4_), respectively, after 2000 cycles.

The energy and power density of CrGOs are plotted in Fig. S32 (ESI†), with: (1) the highest energy density of 29.3 W h kg^−1^ and (2) the highest power density of 10 kW kg^−1^ achieved for rGO(NaBH_4_)_12 h. The energy density is directly proportional to both the specific capacitance and the voltage window. Since the voltage window remains constant in all cases, the efficiency of the various reducing agents and reaction times correlates with the obtained capacitance in a similar trend. The energy and power density values mentioned are highly suitable for applications related to energy storage.^63^ Furthermore, it has been observed that the wettability, which is closely linked to the quantity of OFGs on the surface of rGO samples, plays a significant role in energy storage applications.^64^ Improved wettability enhances the effective energy density, as demonstrated in Table S4 and Fig. S33 (ESI†), where the rGO(NaBH_4_) sample, with the highest oxygen content, exhibits both the best wettability and the highest energy density. Surprisingly, the rGO(N_2_H_4_) samples, despite having a lower oxygen content than rGO(AA)_12 h, are more hydrophilic, probably due to the presence of nitrogen heteroatoms. The higher hydrophilicity of the rGO(N_2_H_4_) and rGO(NaBH_4_) samples is also in full agreement with the lower stability of CrGO films deposited on PET substrates.

### Toxicology studies

**Fig. 5:**
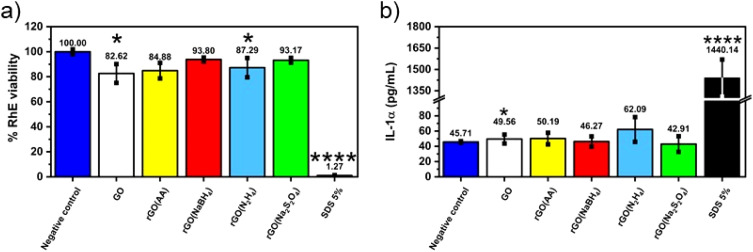
Assessment of skin irritation properties of chemically reduced GOs (a) using the SkinEthic™ Skin Irritation Test^−42bis^ (OCED TG 439). Results represent tissue viability reductions induced by rGOs evaluated using the MTT reduction assay with respect to negative controls (RhE exposed to PBS); 5% SDS was used as the positive control. Results are the mean ± SE of three independent experiments. Statistical differences *vs.* negative controls: *p < 0.05; ****p < 0.0001 (one-way ANOVA and Bonferroni's post-test) and (b) the release of IL-1α from RhE exposed to chemically reduced GOs. IL-1α was measured using a specific sandwich ELISA in growth media collected from RhE after exposure to each material; 5% SDS was used as the positive control. The data, reported as pg mL^−1^ of IL-1α released in the media, are the mean ± SE of three independent experiments. Statistical differences *vs.* negative controls (RhE exposed to PBS): ****p < 0.0001 (one-way ANOVA and Bonferroni's post test).

Given the fact that rGO is highly valued for its application in wearable devices, where exposure to human skin is unavoidable, we conducted toxicology tests specifically aimed at assessing its potential for causing skin irritation, one of the most feasible adverse outcomes at the cutaneous level. The irritation potential of CrGO was determined following the OECD TG 439, using the SkinEthic™ Skin Irritation Test^−42bis^ (42 minutes exposure + 42 hours post-incubation), which is already fruitfully adopted for graphene-related materials.^65^ Briefly, RhE tissues were typically exposed to 16 mg of each material as a powder at the air–liquid interface for 42 minutes followed by 42 hours post-incubation without the materials and the irritation potential was considered when the tissue viability was ≤50%. The MTT reduction assay was utilized to measure the reduction of tissue viability induced by rGOs, as illustrated in Fig. 5. In general, none of the materials’ RhE viability was reduced to levels lower than the threshold value given by the OECD TG 439 (tissue viability ≤50%) and, therefore, they can be considered as non-irritant materials. Among the different samples, the CrGO(AA)_2 h (reduction of cell viability by 15%; *p* < 0.05), rGO(N_2_H_4_)_12 h (reduction of cell viability by 20%; *p* < 0.05) and GO (reduction of cell viability by 17%; *p* < 0.05) were able to slightly reduce RhE viability, even though at levels not predicting the irritant potential. In contrast, the positive control (5% SDS) significantly reduced RhE viability by 99% (*p* < 0.0001), resulting in an irritant compound. The absence of skin irritation was confirmed by the analysis of IL-α release from treated RhE, as an additional biomarker to classify skin irritants using 3D models of the epidermis.^66^ Indeed, despite minor variations, none of the materials significantly increased IL-α release from RhE in contrast to the positive control (1440.1 pg mL^−1^), increasing by more than 30 fold its release with respect to the negative control (45.7 pg mL^−1^). By and large, these results also suggest that the different chemical reductions, possibly leading to chemical reagent residues and/or changes in the material structure, do not appear to affect the good biocompatibility of graphene-related materials previously reported by both *in vitro*^67–72^ and *in vivo*^73,74^ studies.

## Conclusions

In summary, we have introduced an optimized, scalable, easily controlled, and energy-efficient method for producing chemically reduced graphene oxide (CrGO) with on-demand electrical properties. Our findings, corroborated by solid-state NMR (ssNMR), indicate that the peak area corresponding to C_sp^2^_–O species serves as an excellent indicator of the reduction strength of the employed reducing agents. Specifically, we observed the following trend: Na_2_S_2_O_4_ ≈ N_2_H_4_ > AA > NaBH_4_, which aligns with the outcomes of FTIR analysis, elemental analysis (E.A.), and X-ray photoelectron spectroscopy (XPS) analysis. Furthermore, the peak area of the C_sp^2^_–O species revealed the influence of the reaction time on GO reduction for three of the reducing agents, namely Na_2_S_2_O_4_, AA, and NaBH_4_.

Our investigations using E.A. and XPS revealed that the utilization of N_2_H_4_ during the GO reduction process resulted in nitrogen contamination and pyrazole formation. Similarly, NaBH_4_ led to sodium contamination, while AA induced a supramolecular interaction with DHA within the CrGO structure.

Notably, we demonstrated that CrGO with excellent electrical conductivity (>1800 S m^−1^) can be synthesized using AA (with a 12 hour reaction time), Na_2_S_2_O_4_, or N_2_H_4_ (regardless of reaction time). CrGO produced with AA or N_2_H_4_, exhibiting reduced hydrophilicity, enabled the fabrication of films on flexible plastic substrates, maintaining resistance even after enduring 2000 bending cycles.

CrGO synthesized with NaBH_4_ in a 12 hour reaction exhibited superior electrochemical performance, boasting a specific capacitance of 211 F g^−1^ at a current density of 0.5 A g^−1^ and a capacitance retention exceeding 99.5% after 2000 cycles, surpassing the other reducing agents. While the loss of OFGs detrimentally affected electrochemical performance, this drawback was mitigated by a significant increase in surface area, pore size, and ionic conductivity, yielding values of ∼555.63 m^2^ g^−1^, 9.60 nm, and 1.04 S m^−1^, respectively.

Furthermore, skin irritation tests demonstrated that all variants of CrGO could be considered non-irritating materials, affirming that the reduction process does not compromise their biocompatibility at the cutaneous level. These findings represent a significant advancement in the application of reduced graphene oxide (rGO), particularly in wearable and flexible electronics with skin-level interactions.

## Conflicts of interest

There are no conflicts to declare.

## Supplementary Material

NR-015-D3NR04521H-s001
