# Supplementary material for: A comparative investigation of the chemical reduction of graphene oxide for electrical engineering applications
Source: Nanoscale. 2023 Oct 20;15(44):17765–75. doi: 10.1039/d3nr04521h (PMC10653029; doi:10.1039/d3nr04521h)
Supplement: NR-015-D3NR04521H-s001 [file NR-015-D3NR04521H-s001.pdf]

Electronic Supplementary Material (ESI) for Nanoscale.
This journal is © The Royal Society of Chemistry 2023

# Electronic Supplementary Information

## A comparative investigation of chemical reduction of graphene oxide for electrical engineering applications

Tomasz Chudziak,[a,b] Verónica Montes-García,[c] Włodzimierz Czepa,[a,b] Dawid Pakulski,[b] Andrzej Musiał,[b,d] Cataldo Valentini,[b] Michał Bielejewski,[d] Michela Carlin,[e] Aurelia Tubaro,[e] Marco Pelin,[e] Paolo Samorì*[c] and Artur Ciesielski*[b,c]

[a] *Faculty of Chemistry Adam Mickiewicz University, Uniwersytetu Poznańskiego 8 Poznań, Poland*

[b] *Center for Advanced Technologies Adam Mickiewicz University, Uniwersytetu Poznańskiego 10 Poznań, Poland*

[c] *Université de Strasbourg CNRS, ISIS, UMR 7006, 8 Allée Gaspard Monge, F-67000 Strasbourg, France*

[d] *Institute of Molecular Physics, Polish Academy of Sciences, M. Smoluchowskiego 17, 60-179 Poznań, Poland*

[e] *University of Trieste, Department of Life Sciences, Via Fleming 22, 34127 Trieste, Italy*

## Table of Contents

**Section A. Materials and characterization** ........ S2

**Section B. Physical characterization** ........ S15

- **Fourier-transform infrared (FTIR) spectroscopy** ........ S15
- **Elemental analysis (EA)** ........ S16
- **X-ray Photoelectron Spectroscopy (XPS)** ........ S17
- **Solid-state MAS NMR** ........ S22
- **Raman spectroscopy** ........ S25
- **Powder X-Ray Diffraction (PXRD)** ........ S27
- **Scanning electron microscopy (SEM)** ........ S28
- **Thermogravimetric analysis (TGA)** ........ S29
- **BET characterization** ........ S30

**Section C. Electrochemical characterization** ........ S36

**References** ........ S43

**Section A. Materials and characterization**

**Experimental details**

**Materials:** L-ascorbic acid (AA), sodium borohydride ($NaBH_4$), hydrazine monohydrate ($N_2H_4$), sodium hydrosulfite ($Na_2S_2O_4$), 25 wt% ammonia solution ($NH_3$), potassium carbonate ($K_2CO_3$), ethanol. All of the reductors were bought from Sigma-Aldrich and used directly without further purification. Gaphene Oxide (GO) water dispersion with concentration 1 wt% was acquired from Graphenea, Separators Whatman® glass microfiber filters, binder poly(tetrafluoroethylene) and 1-methyl-2-pyrrolidinone were purchased from Sigma Aldrich. Conductive Carbon Black Super P (H30253) was acquired from Alfa Aesar and carbon AvCarb P75 substrate was gained from FuelCellStore.

**GO films.** GO water solution (0.4 mg/mL) was spray coated onto polyethylene terephthalate (PET) substrates at 90°C (2.5×1.2 $cm^2$).

**General reduction procedure:** An aqueous dispersion of 30 mL of GO 10 mg/mL was diluted in 270 mL of Milli Q water and sonicated for 20 minutes in an ultrasonic bath cleaner (140 w). Subsequently, the reducing reagent was added and the pH adjusted to 9-10. The reaction mixture was stirred for a certain amount of time (see Table S1) at 95 °C. The chemically-reduced GO (CrGO) was collected by filtration and washed thoroughly with deionized water and ethanol. The black precipitate was then freeze-dried for 72 h under vacuum.

**Table S1.** Synthetic conditions studied for the preparation of CrGO.

| **Reducing agent** | **Concentration (g/L)** | **pH adjusting reagent** | **Reaction time (h)** | **Reaction temperature (ºC)** |
|---|---|---|---|---|
| $NaBH_4$ | 8 | 5 wt% $K_2CO_3$ | 2 | 95 |
| $NaBH_4$ | 8 | 5 wt% $K_2CO_3$ | 12 | 95 |
| Ascorbic Acid | 8 | 25 wt% $NH_3$ | 2 | 95 |
| Ascorbic Acid | 8 | 25 wt% $NH_3$ | 12 | 95 |
| $N_2H_4$ | 10 | - | 2 | 95 |
| $N_2H_4$ | 10 | - | 12 | 95 |
| $Na_2S_2O_4$ | 7.5 | 25 wt% $NH_3$ | 2 | 95 |
| $Na_2S_2O_4$ | 7.5 | 25 wt% $NH_3$ | 12 | 95 |

**Reduction of GO films**: GO films were immersed in 30 mL aqueous solution of the reducing agent at the desired concentration (dilution 1:10 of the amount indicated in Table S1) for 2 hours.

**Methods:** Structural data were obtained by **X-Ray diffraction (XRD)** with the use of Bruker AXS D8 Advance diffractometer in Bragg-Brentano geometry with Cu$K_{\alpha 1}$ radiation ($\lambda$ = 1.5406 Å). **X-ray Photoelectron Spectroscopy (XPS)** analyses were performed with a Thermo Scientific $K_{\alpha}$ X-ray Photoelectron Spectrometer system equipped with an Al $K_{\alpha}$ X-Ray source (photon energy $E_{ph}$ = 1486.6 eV, beam spot size ~100 μm. For the **nuclear magnetic resonance (NMR) spectroscopy** $^{1}$H and $^{13}$C spectra were recorded on a Bruker Avance III HD spectrometer coupled to an 11.7 T wide bore superconducting magnet operating at 500 MHz $^{1}$H Larmor frequency and 125.76 MHz $^{13}$C Larmor frequency. All spectra were recorded at 298K stabilized temperature using the magic-angle spinning technique for high-resolution NMR spectroscopy in solid-state using 4mm zirconia rotors. The spinning frequency was equal to 12 kHz for $^{1}$H and $^{13}$C nuclei. A speed synchronized spin-echo was included inside the experimental pulse sequences for the $^{1}$H and $^{13}$C spectra, recoded with direct polarization to get undistorted line shapes and filter out background probe signals. The echo time was kept identical for all $^{1}$H and $^{13}$C spectra and equal one rotation period. The total echo time was 83 μs, the recycle delay was 5 s, and the number of scans was 512 for proton spectra. Due to the very high conductivity of studied samples, no cross-polarization experiment was possible, owning to non-effective energy transfer. Therefore, the $^{13}$C spectra were recorded using the direct polarization method with recycle delay equal to 10 s and 8192 scans, leading to almost 23 hours of data accumulation per spectrum. **Elemental analysis (EA)** was performed on an Elementar Analyser Vario EL III. Pore-size distribution and specific surface area of (Cr)GO samples were investigated by the nitrogen adsorption–desorption isotherm (Autosorb iQ, Quantachrome) using **Barrett-Joyner-Halenda (BJH)** and **Bruanauer-Emmett-Teller (BET)** techniques, respectively. **Scanning electron microscopy (SEM)** was performed on an FEI Quanta 250 FEG Scanning Electron Microscope operating at an accelerating voltage of 5 keV incident beam energy. **Raman** spectra were performed using a Renishaw InVia Reflex system. The spectrograph used a high-resolution grating (2400 grooves $cm^{-1}$) with additional bandpass filter optics, a confocal microscope, and a 2D-CCD camera. The excitation was carried out using a 532 nm laser excitation beam, with a 100× objective, 0.2 mW maximum power and 1 s acquisition time. **Thermogravimetric analysis (TGA)** was carried out in the

temperature range 30–1000 °C operating under ambient conditions, with a ramp rate of 10 °C $min^{-1}$ on a Mettler Toledo TGA/SDTA851e system. **Fourier transform infrared (FTIR)** spectra were recorded within the mid-IR range (500–4000 $cm^{-1}$) using a PerkinElmer spectrometer (spectrum two) equipped with ATR Diamond.

**Four-Point Probe measurements.** Electrical conductivity measurements were conducted on pelletized samples: 50 mg of GO or CrGO were pressed under 10 tons with a Specac press machine. Films electrical resistivity were measured with Jandel, Model RM3000, limit of detection $10^7$ $Wsq^{-1}$. The resistivity (r) was obtained

$$\rho = R_s \cdot l \tag{1}$$

Where *Rs* is the sheet resistance and *l* is thickness of the film.

**Calculation of the average defect distance ($L_D$):**

$$L_D^2(nm^2) = (1.8 \pm 0.5) \times 10^{-9} \lambda_L^4 \left(\frac{I_D}{I_G}\right)^{-1} \tag{2}$$

Where, $\lambda_L$ is the laser wavelength (nm)

**Calculation of defect density ($n_D$):**

$$n_D(cm^{-2}) = \frac{(1.8 \pm 0.5) \times 10^{22}}{\lambda_L^4} \left(\frac{I_D}{I_G}\right) \tag{3}$$

**Fabrication of supercapacitors.** The electrochemical performance of the chemically reduced graphene oxide was measured in a two-electrode symmetric supercapacitor system. Two electrodes were assembled in CR2032 stainless steel coin-type cells with a Whatman® glass microfiber filters as a separator and 1 M $H_2SO_4$ aqueous solution as electrolyte.

The working electrodes were prepared by mixing of given rGO sample (80% wt%, 8 mg), carbon black (10wt%, 1 mg) and PTFE (10wt%, 1 mg) with 1 ml of NMP, sonicated for 20 mins and subsequently droplet deposited on carbon substrate placed on hot plate (80° C) to quickly evaporate the solvent achieving homogenously covered electrode. The electrodes were

additionally dried under vacuum at 80° C (24h). The mass loading of the (chemically-reduced) graphene oxide was ~2-3 mg in each electrode.

The devices were electrically characterized by cyclic voltammetry (CV), galvanostatic charge/discharge (GCD) and electrochemical impedance spectroscopy (EIS) employing a EC-LAB VMP3 (BioLogic Science Instruments). CV was performed in the potential window from 0 to 1 V at scan rates ranging from 2 mV/s to 1000 mV/s. GCD curves were recorded in the same potential window at different current densities (1-10 A/g). The frequency range for the impedance spectra was from 100 kHz to 1 mHz with a sine-wave voltage signal amplitude of 10 mV (root-mean-square, RMS).

**Calculation of the specific capacitances, energy densities and power densities.** From charge-discharge measurements, the specific capacitances (Cp), energy densities (E) and power densities (P) of (chemically reduced) graphene oxide were obtained from the acquired data using following equations[1]:

$$Cp = \frac{2 \cdot I \cdot \Delta t}{m \cdot \Delta V} \tag{4}$$

$$E = \frac{1}{2} \cdot Cp \cdot \Delta V^2 \tag{5}$$

$$P = \frac{E}{\Delta t} \tag{6}$$

where I is the discharge current (A), Δt is the discharge time (s), m is the weight of the active material in an individual electrode (g), and ΔV is the discharge voltage (V) excluding the internal iR drop during the discharge process.

**Calculation of ionic conductivity (σ)**

$$\sigma = \frac{l}{R_i \cdot A} \tag{7}$$

Being l the film thickness (100 μm), A the film area (1.27 $cm^2$) and Ri is the ionic resistance.

**Toxicological analysis**

**Evaluation of skin irritation:**

SkinEthic™ Reconstructed Human Epidermis (RhE, provided by EpiSkin; Lyon, France), was used for evaluating skin irritation potential of rGOs, following the OECD Test Guideline (TG) No. 439. According to the TG, before being used for the experiments, RhE tissues were checked for quality control criteria (mean optical density, O.D., of 3 negative controls = 1.15 ± 0.05; mean viability of 3 positive controls, 5% SDS = 1.3 ± 0.1 %; exposure time inducing 50 % viability using Triton X-100 1 %, ET50 = 8.7 ± 0.3 h), that were all within the acceptance range. Technical proficiency was assessed testing the ten proficiency substances listed in Annex 3 of the OECD TG 439, as previously reported.[2] In addition, unspecific interaction of rGOs with the MTT readout was preliminary evaluated on "killed" RhE, obtained by freezing treatment at -80°C for 48 h and none of the materials significantly increased MTT conversion, suggesting no unspecific interactions for rGOs.

The assessment of irritation potential of rGOs on SkinEthic™ RhE model was performed following the skin Irritation Test[-42bis], in compliance with the OECD TG 439, as previously reported for other graphene-related materials (Fusco et al., 2020). Briefly, after being wetted with 10 μL of distilled water, RhE tissues (dimensions of 0.5 $cm^2$, at day 17) were topically exposed to 16 mg of each rGO (concentration of 32 mg $cm^{-2}$) in triplicate for 42 min at room temperature (RT). As negative and positive controls, RhE tissues were exposed to phosphate buffered saline (PBS) or 5% w/v sodium dodecyl sulphate (SDS), respectively. After exposure, RhE tissues were washed 25 times with 1 mL PBS and transferred in a 6-well plate with 2 mL growth medium for 42 h at 37 °C and 5% $CO_2$. Tissue were then transferred in a 24-well plate containing 300 μL of MTT solution (1 mg $mL^{-1}$) for 3 h at 37 °C and 5% $CO_2$ and the resulting formazan salts were extracted with isopropanol (1.5 mL per well, for 2 h at RT) and measured at 570 nm using the FLUOstar® Omega microplate reader (BMG LABTECH, Ortenberg, Germany). Tissue viability is reported as % of negative controls and are the mean ± standard error (SD) of three independent experiments.

**Interleukin-1α quantitation**

After 42 minutes of exposure to rGO or positive control followed by 42 hours of post-incubation time, culture media were collected and stored at -80 °C. Interleukin (IL)-1α was quantified

using a specific sandwich ELISA kit from Diaclone (Tema Ricerca, Milan, Italy) following the producer's instructions. Results are expressed as pg/mL of IL-1α released by the tissues in the growth medium and are the mean ± SD of three independent experiments.

## Statistical analysis

For skin irritation (OECD TG 439), the results are expressed as % of tissue viability with respect to negative controls and are the mean ± SD of three independent experiments. As a threshold given by OECD TG 439, viability ≤ 50 % defines an irritant material.

Statistical analysis was performed by a one-way ANOVA followed by Bonferroni's post-test (GraphPad Prism version 8.00) and statistical significance was considered for $p < 0.05$.

**Table S2.** State of the art of the conditions to obtain chemical reduced graphene oxide, their structural characteristics and their electrical performances.

| Reducing agent | Concentration Reducing agent [g/L] | Concentration GO [g/L] | pH adjusting reagent | Reaction time [h] | Reaction temperature [°C] | C/O ratio XPS | Electrical conductivity [S/m] | Surface area BET [m$^2$/g] | Capacitance [F/g] | Ref. |
|---|---|---|---|---|---|---|---|---|---|---|
| $NaBH_4$ | **8** | **1** | **5 wt% $K_2CO_3$** | **2** | **95** | **4.64** | **27** | **442.53 Pore size: 3.20 nm** | **86 F/g at 0.5 A/g, 1M $H_2SO_4$, two-electrode configuration** | **This work** |
| | | | | **12** | | **4.76** | **41** | **555.63 Pore size: 9.60 nm** | **211 F/g at 0.5 A/g, 1M $H_2SO_4$, two-electrode configuration** | |
| | 7 | 1 | 6 M KOH/ ammonia solution | 8 | 95 | 10.00 | 48 | - | 126.4 F/g at 1 A/g 6M KOH three-electrode configuration | [3] |
| | 0.57 | 0.5 | - | 4 | RT | 1.65 | - | - | 1.1 F/g at 0.05 A/g PVA/$H_3PO_4$ three-electrode configuration | [4] |
| | 1.89 | | | | | 1.65 | | | 1.5 F/g at 0.05 A/g PVA/$H_3PO_4$ three-electrode configuration | |
| | 3.78 | | | | | 1.59 | | | 5.9 F/g at 0.05 A/g PVA/$H_3PO_4$ three- | |

| | | | | | | | | | | |
|---|---|---|---|---|---|---|---|---|---|---|
| | | | | | | | | | electrode configuration | |
| | 1.89 | | | | 105 | 4.04 | | | 30.1 F/g at 0.05 A/g PVA/$H_3PO_4$ three-electrode configuration | |
| | 10 | 1 | 5 M KOH (5mL) | 24 | 180 | - | | 290.35 Pore volume 0.4 $cm^3/g$ Pore size 4.82 nm | 183 F/g at 1 A/g 1 M $H_2SO_4$ three-electrode configuration | 5 |
| | $NaBH_4$ to GO (10:1) (*m/m*) | - | - | 8 | 98 | - | | 164.13 Pore size 7.18 nm | 120 F/g at 1 A/g 1M $Et_4NBF_4$/PC two-electrode configuration | 6 |
| Ascorbic acid (AA) | **8** | **1** | **25 wt% $NH_3$** | **2** | **95** | | **292** | **292.84 Pore size: 6.39 nm** | **55 F/g at 0.5 A/g, 1M $H_2SO_4$, two-electrode configuration** | **This work** |
| | | | | **12** | | | **1851** | **394.90 Pore size: 13.70 nm** | **121 F/g at 0.5 A/g, 1M $H_2SO_4$, two-electrode configuration** | |
| | 0.1 | 0.1 | - | 48 | RT | - | 800 | - | - | 7 |
| | 12 | 4 | - | 16 | 40 | - | 100 | 512 Pore volume 2.48 $cm^3/g$ | 128 F/g at 50mA/g 6 M KOH two-electrode configuration | 8 |
| | 0.35 | 0.1 | 25% ammonia solution (2μL | 0.25 | 95 | 0.08 (O/C) | 7700 | - | - | 9 |

| | | | | | | | | | | |
|---|---|---|---|---|---|---|---|---|---|---|
| | | | per mL) | | | | | | | |
| | 1 | 0.1 | 25% ammonia solution (20µL per mL) | 2 | 95 | 5.15 | 980 | - | - | 10 |
| | AA to GO (1:5) (*m/m*) | - | - | 4 | 90 | - | 220 | 81 Pore volume 1.22 $cm^3/g$ | 79 F/g at 1 A/g 2 M KOH three-electrode configuration | 11 |
| | 3 | 0.1 | - | 48 | RT | 3.5 | - | - | 63.5 F/g at 100 mV/s 1 M $H_2SO_4$ three-electrode configuration | 12 |
| | AA to GO (10:1) (*m/m)* | - | - | 24 | 80 | - | - | 240.97 Pore size 5.17 nm | 133 F/g at 1 A/g 1M $Et_4NBF_4$/PC two-electrode configuration | 6 |
| AA/$NaHSO_3$ | AA to $NaHSO_3$ (1:1) Reductant to GO (1:5) (*m/m*) | - | - | 4 | 90 | - | 360 | 135 Pore volume 2.9 $cm^3/g$ | 165 F/g at 1 A/g 2 M KOH three-electrode configuration | 11 |
| Hydrazine hydrate | **10** | **1** | **-** | **2** | **95** | | **1857** | **96.41 Pore size: 5.20 nm** | **57 F/g at 0.5 A/g, 1M $H_2SO_4$, two-electrode configuration** | **This work** |
| | | | | **12** | | | **3848** | **124.92 Pore size: 6.4 nm** | **92 F/g at 0.5 A/g, 1M $H_2SO_4$, two-electrode** | |

| | | | | | | | | | | |
|---|---|---|---|---|---|---|---|---|---|---|
| | | | | | | | | | **configuration** | |
| | 5mL (80 wt %) | 100mg | - | 72 | RT | - | - | 320 | 205 F/g at 100 mA/g 30 wt % KOH two-electrode configuration | 13 |
| | 1:5, GO/hyrdrazine | 2 | - | 24 | 95 | 14.11 | 1980 | - | 84.7 F/g at 1 A/g 2 M $H_2SO_4$ three-electrode configuration | 14 |
| | 10 | 1 | ammonia solution | 8 | 95 | 11.74 | 76 | - | 152.5 F/g at 1 A/g 6M KOH three-electrode configuration | 3 |
| Sodium dithionite | **7.5** | **1** | **25 wt% $NH_3$** | **2** | **95** | | **3369** | **140.91 Pore size: 1.4 nm** | **37 F/g at 0.5 A/g, 1M $H_2SO_4$, two-electrode configuration** | **This work** |
| | | | | **12** | | | **4324** | **278.34 Pore size: 7.30 nm** | **106 F/g at 0.5 A/g, 1M $H_2SO_4$, two-electrode configuration** | |
| | 12.5 | GO film | NaOH | 0.25 | 60 | - | 1377 | - | - | 15 |
| | 7.5 | 1 | 25 wt% $NH_3$ | 12 | 90 | 6.6 | - | - | - | 16 |
| Hydroxylamine ($NH_2OH$) | 0.5 | 0.25 | ammonia aqueous solution (30% in volume) 200µL | 0 | 90 | 1.9:1 | - | - | - | 17 |
| | | | | 0.5 | | 6.1:1 | - | | | |
| | | | | 1 | | 9.7:1 | 1122 | | | |
| Liquid borane-THF adduct | 1.5mL | 3 | - | 96 | 100 | 7.8 | 50 | 466 Pore size 2.13nm | 200 F/g at 0.1A/g 6M KOH | 18 |

| | | | | | | | | | | |
|---|---|---|---|---|---|---|---|---|---|---|
| | | | | | | | | | two-electrode configuration<br>193 F/g at 0.1 A/g 6M KOH three-electrode configuration | |
| *Hibiscus sabdariffa L.* aqueous extract | 200mL of extract | 0.4 | 0.01M $H_2SO_4$ | 1 | At refluxed [100] | 3.0 | - | - | 41 F/g at 100 mV/s 133.07 at 5 mV/s 1 M $H_2SO_4$ two-electrode system | 19 |
| Pyrogallol | 1mM | 0.1 | 25% ammonia solution (2µL per mL) | 1 | 95 | 0.18 (O/C) | 488 | - | - | 9 |
| HBr | 3mL (40%) | 0.1 | - | 24 | 110 | 3.9 | 0.023 | - | 129 F/g at 10 mV/s 1 M $H_2SO_4$ two-electrode 348 F/g at 0.2A/g 1 M $H_2SO_4$ two-electrode 158 F/g at 0.2A/g 1 M $BMIPF_6$ two-electrode<br>171 F/g at 10 mV/s 1 M $H_2SO_4$ three-electrode configuration | 20 |
| HI | HI vapours | Spin coated | - | 0.08 | 90 | - | 831.35 | - | - | 21 |

| | | | | | | | | | | |
|---|---|---|---|---|---|---|---|---|---|---|
| | | layer | | | | | | | | |
| | HI (45%) | | - | 2 | | 7.1 | - | | 180 F/g at 10 mV/s 1 M $H_2SO_4$ three-electrode configuration | 22 |
| L-serine | 3 | 1 | NaOH/HCl diluted solutions | 10 | 90 | 7.9 | - | - | - | 23 |
| L-cysteine | 10 | 0.5 | - | 72 | RT | - | 0.124 | - | - | 24 |
| D-glucose | 3 | 0.1 | 25% ammonia solution | 2 | 95 | 2.89 | 315 | - | - | 10 |
| Gallic acid | 40 | 4 | ammonia aqueous solution 1.2mL | 24 | RT | 3.89 | 0.96 | - | - | 25 |
| | | | | 6 | 95 | 5.28 | 36 | | | |
| KOH/ hydrazine hydrate | 1:5 (*m/m*) GO/hyrdrazine | 2 | - | 24 | 95 | 10.13 | 431 | - | 253 F/g at 0.2 A/g 2 M $H_2SO_4$ three-electrode configuration | 14 |
| Thiourea dioxide | 28 | 5 | NaOH | 1 | 90 | 14.5 | - | - | - | 26 |
| | | | | 2 | | 16.0 | | | | |
| Sn - powder | 5 | 1 | HCl (35%) 20mL | 3 | 40 | 6.1 | 8.650 | - | 152 F/g at 1.5 A/g 1 M $H_2SO_4$ three-electrode configuration | 27 |
| Zn - powder | Zn powder scattered 74µL | 1 | 25% ammonia solution | 0.16 | RT | 8.05 | 2.160 | - | 116 F/g at 0.05 A/g 6 M KOH three-electrode configuration | 28 |

| | | | | | | | | | | |
|---|---|---|---|---|---|---|---|---|---|---|
| Al - powder | 5 | 1 | 0.5 M HCl 5mL | 0.5 | RT | 18.6 | 2.100 | 365 | - | [29] |

## Section B. Physical characterization

### Fourier-transform infrared (FTIR) spectroscopy

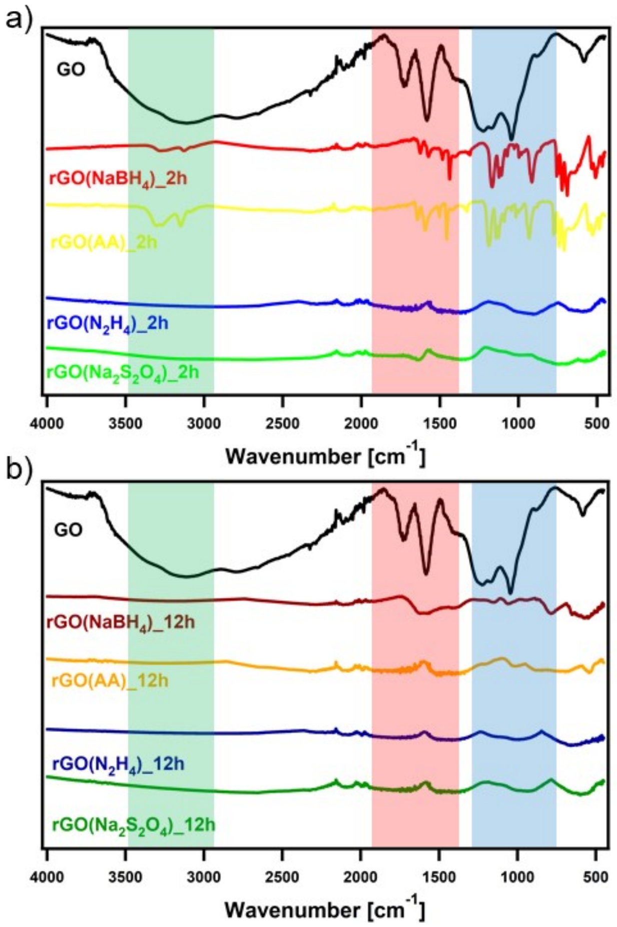


**Figure S1.** FTIR spectra of chemically reduced GO with different reducing agents at a) 2 hours and b) 12 hours reaction time.

**Elemental analysis (EA)**

**Table S3.** Elemental analysis of chemically reduced GO samples.

| Sample | %C | %O | %N | %S | %H | C/O |
|---|---|---|---|---|---|---|
| GO | 46.10 | 46.60 | 0.03 | 2.00 | 5.27 | 0.99 |
| rGO($NaBH_4$)_2h | 65.00 | 31.94 | 0.17 | 1.17 | 1.73 | 2.04 |
| rGO($NaBH_4$)_12h | 66.72 | 30.29 | 0.58 | 1.11 | 1.30 | 2.20 |
| rGO(AA)_2h | 75.51 | 20.59 | 1.99 | 0.88 | 1.03 | 3.67 |
| rGO(AA)_12h | 70.74 | 26.32 | 0.97 | 0.92 | 1.05 | 2.69 |
| rGO($N_2H_4$) _2h | 80.28 | 14.62 | 3.35 | 1.01 | 0.74 | 5.49 |
| rGO($N_2H_4$)_12h | 83.50 | 11.60 | 3.63 | 0.74 | 0.54 | 7.20 |
| rGO($Na_2S_2O_4$)_2h | 81.47 | 14.89 | 0.72 | 2.16 | 0.77 | 5.47 |
| rGO($Na_2S_2O_4$)_12h | 84.78 | 12.07 | 0.41 | 2.09 | 0.65 | 7.02 |

## X-ray Photoelectron Spectroscopy (XPS)

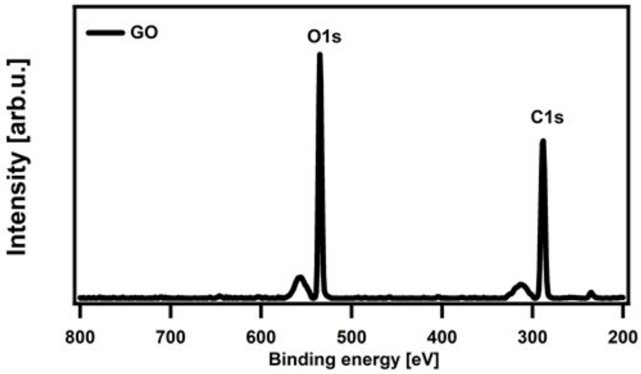


**Figure S2.** Survey spectra of GO.

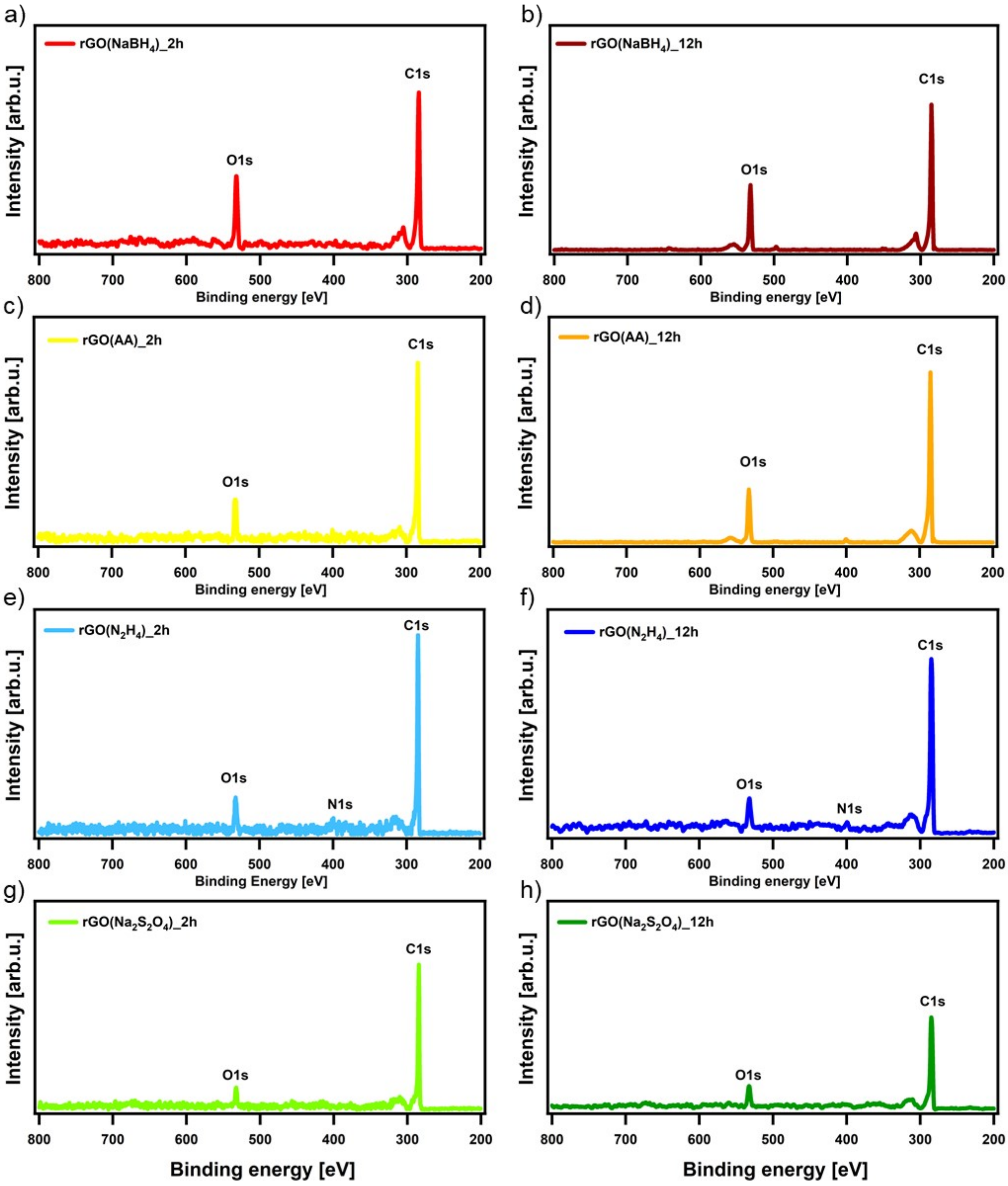


**Figure S3.** Survey spectra of chemically reduced GO with different reducing agents and different reaction times.

**Table S4.** Elemental analysis of chemically reduced GO samples from XPS survey.

| Sample | %C | %O | %N | %Na | C/O |
|---|---|---|---|---|---|
| GO | 46.10 | 53.87 | 0.03 | - | 0.86 |
| rGO($NaBH_4$)_2h | 81.12 | 17.50 | - | 1.37 | 4.64 |
| rGO($NaBH_4$)_12h | 82.10 | 17.25 | - | 0.65 | 4.76 |
| rGO(AA)_2h | 87.37 | 12.63 | - | - | 6.92 |
| rGO(AA)_12h | 87.80 | 12.20 | - | - | 7.20 |
| rGO($N_2H_4$) _2h | 89.79 | 8.45 | 1.76 | - | 10.63 |
| rGO($N_2H_4$)_12h | 88.50 | 8.60 | 2.90 | - | 10.29 |
| rGO($Na_2S_2O_4$)_2h | 91.39 | 8.61 | - | - | 10.62 |
| rGO($Na_2S_2O_4$)_12h | 91.81 | 8.19 | - | - | 11.21 |

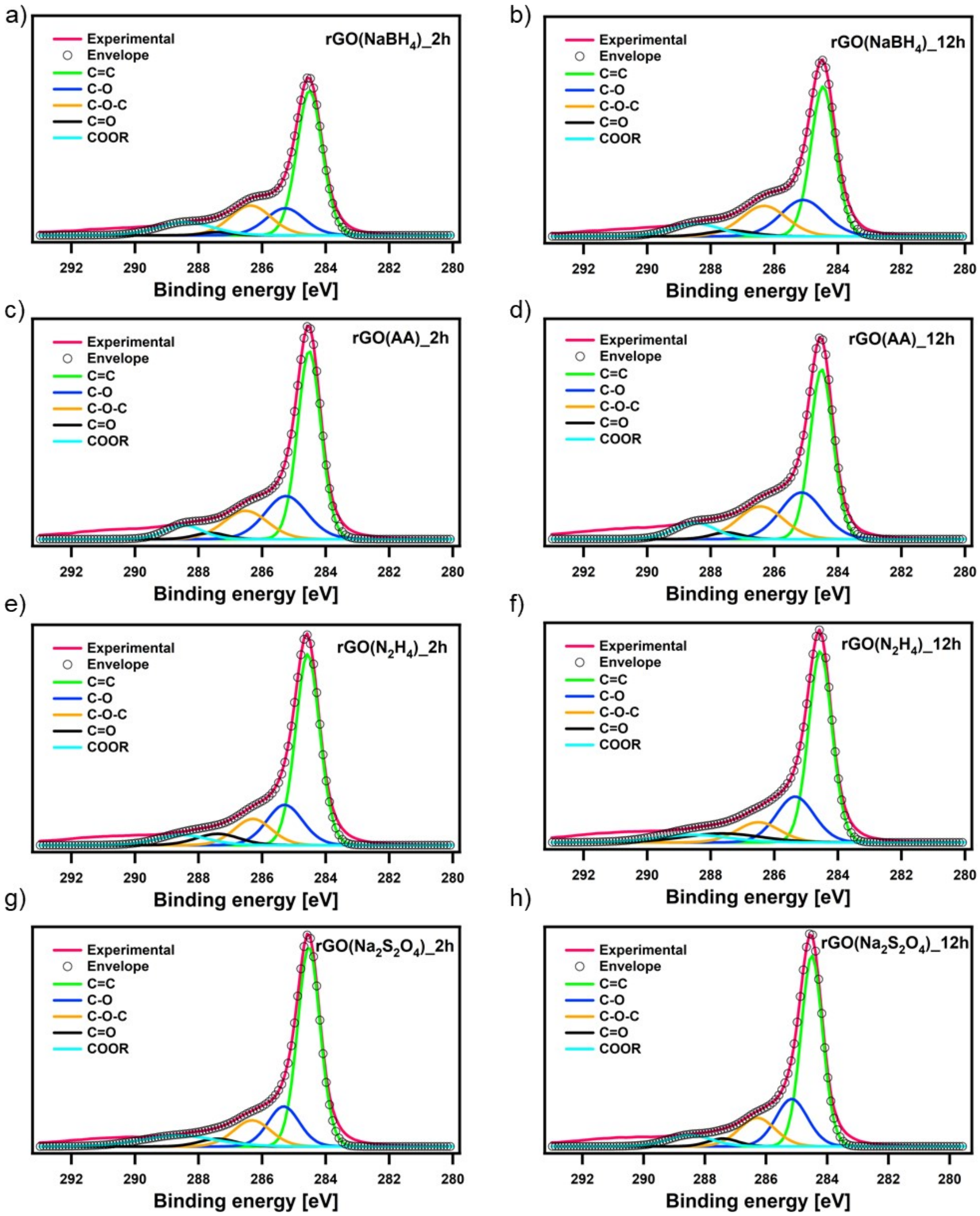


**Figure S4.** High-resolution XPS spectra of C1s for the CrGO with different reducing agents and different reaction times.

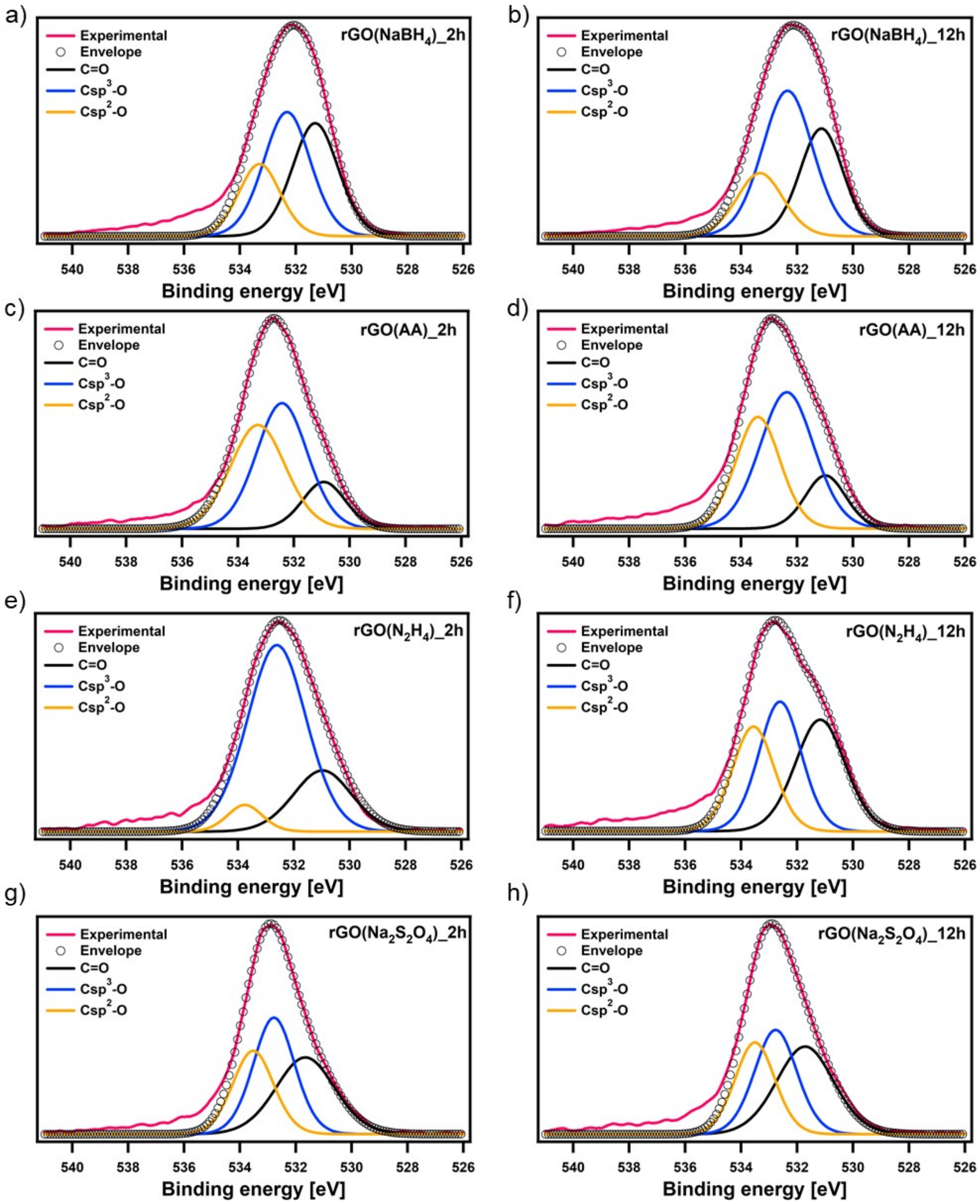


**Figure S5.** High-resolution XPS spectra of O1s for the CrGO with different reducing agents and different reaction times.

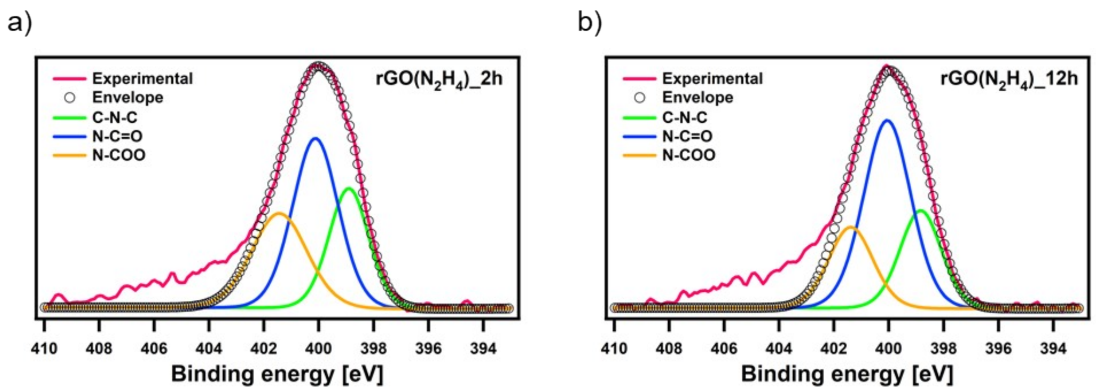


**Figure S6.** XPS analysis of 1Ns spectra of CrGO with hydrazine at a) 2 and b) 12 hours of reduction.

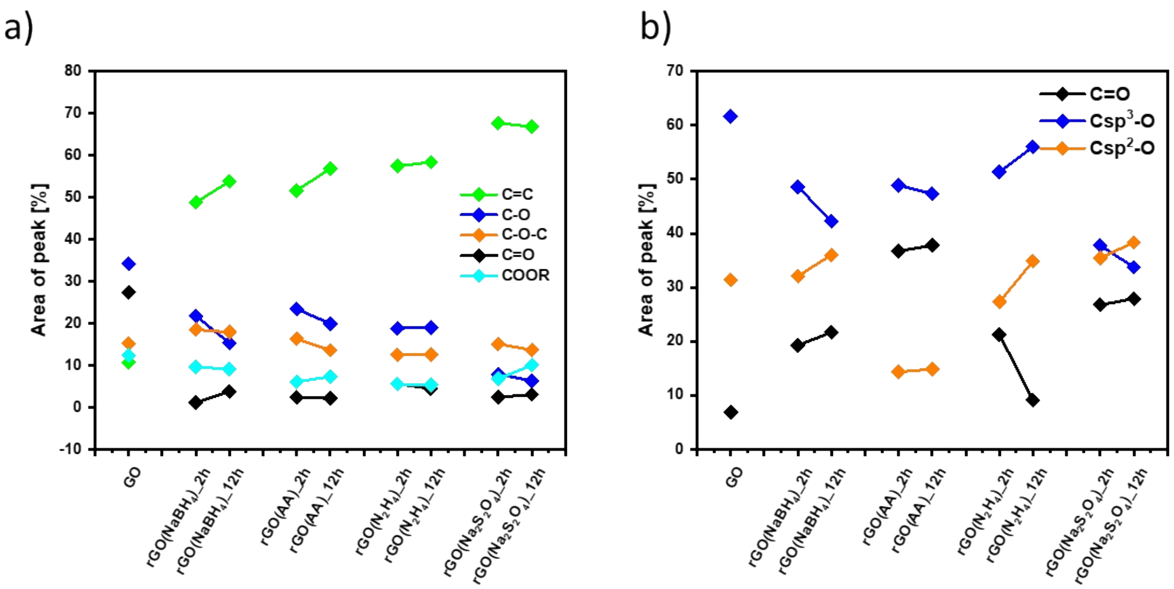


**Figure S7.** a-b) relative contribution of a) C1s peak and b) O1s peak components estimated by dividing the area under each component by whole a) C1s peak area and b) O1s peak area as a function of the reducing agents and reaction time.

## Solid-state MAS NMR

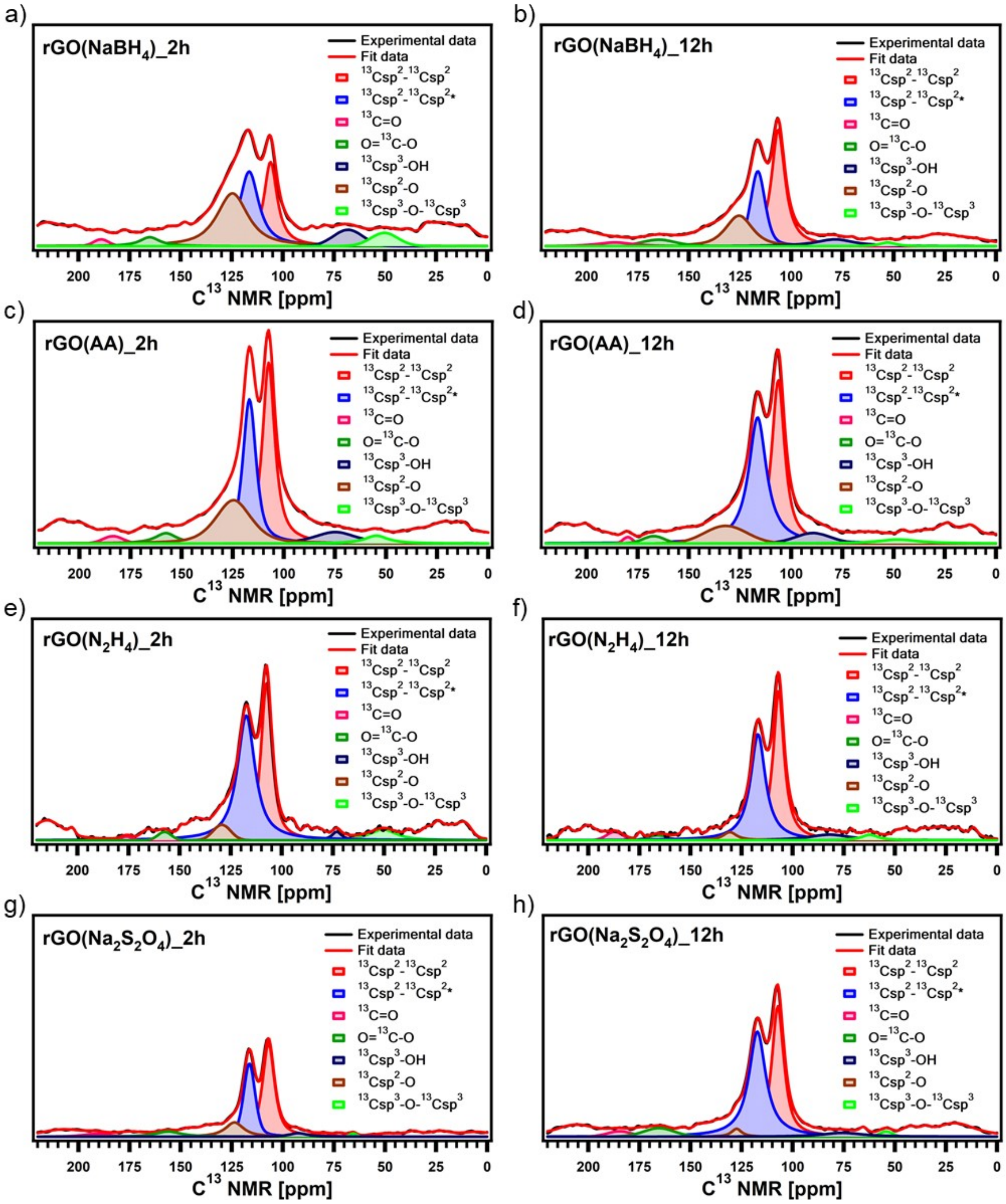


**Figure S8.** Solid-state MAS-NMR C1s spectra of chemically reduced GO.

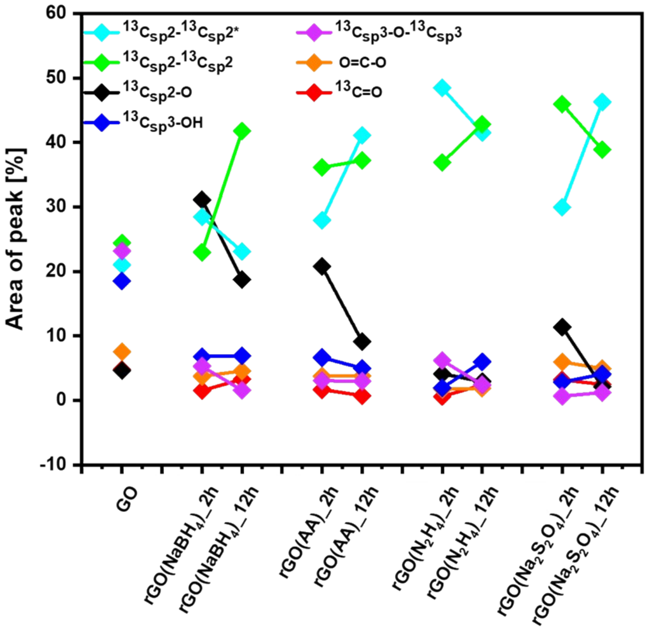


**Figure S9.** Relative contribution of NMR spectra components as a function of the reducing agents and reaction time estimated by dividing the area under each component by the whole peak area.

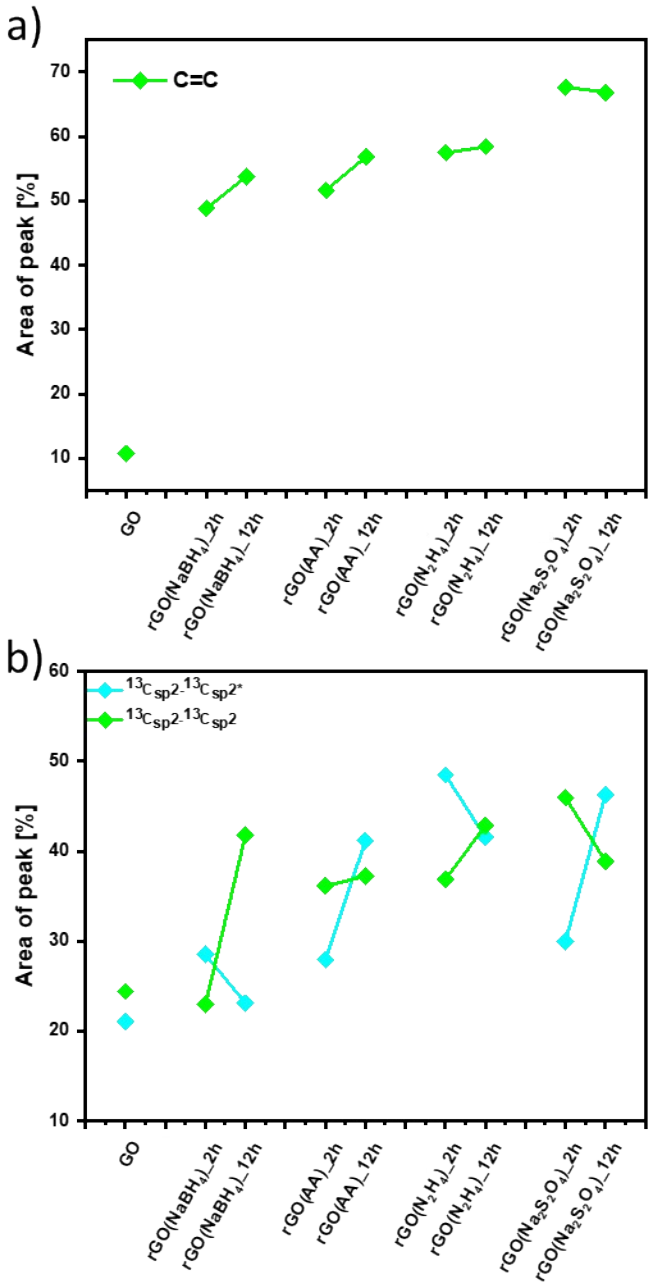


**Figure S10.** Comparison of the relative contribution of a) C1S XPS and b) NMR spectra components as a function of the reducing agents and reaction time estimated by dividing the area under each component by the whole peak area.

**Raman spectroscopy**

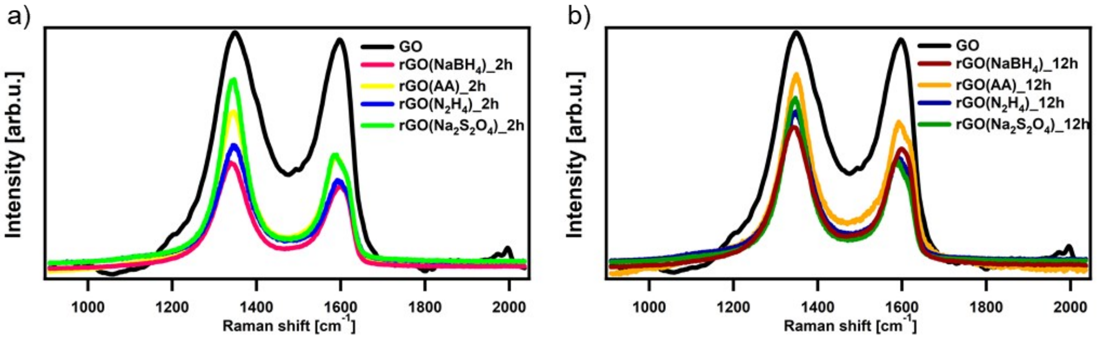


**Figure S11.** Overlapped Raman spectra of GO and CrGO with different reducing agents at a) 2 and b) 12 hours of reaction time.

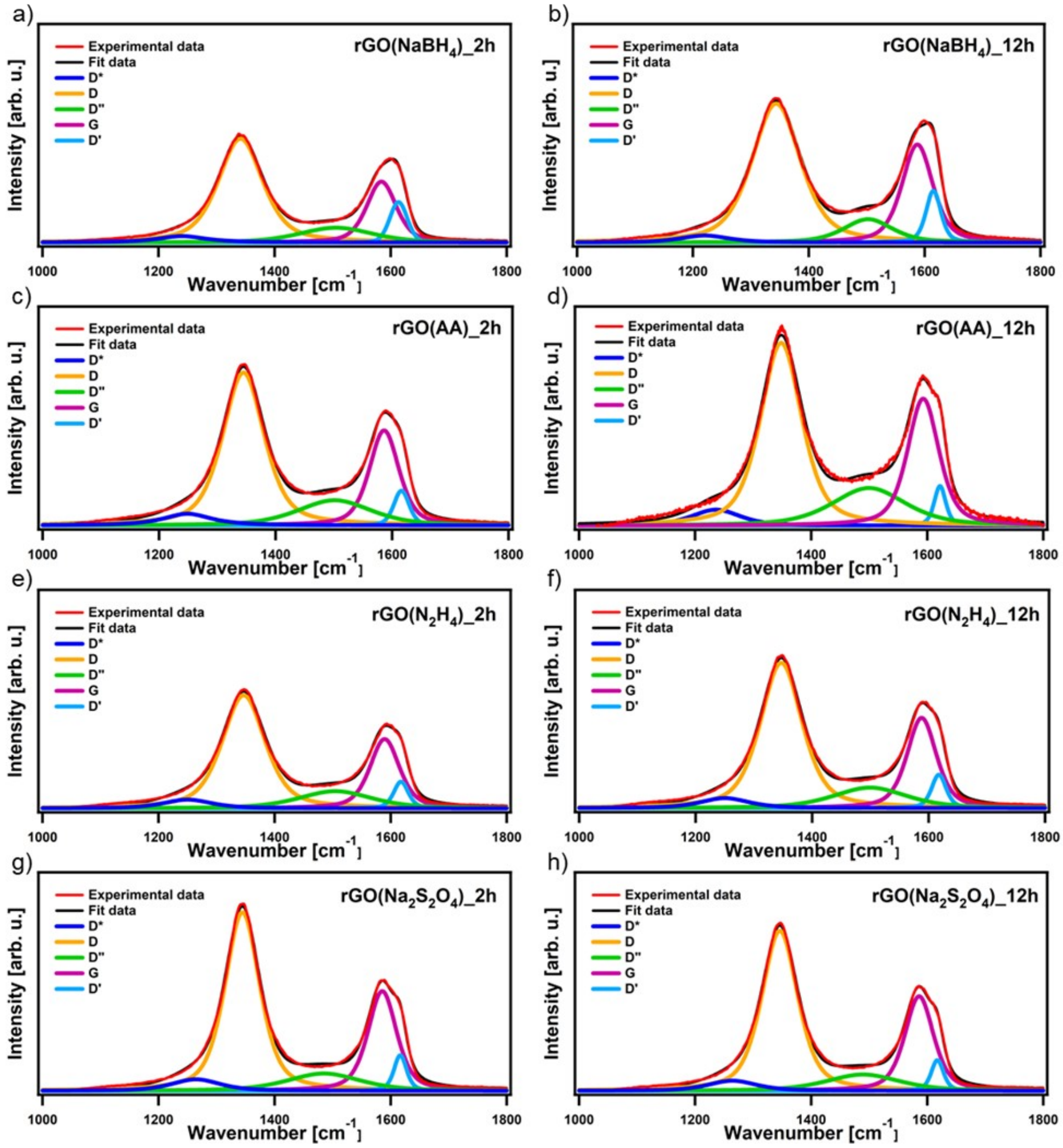


**Figure S12.** Fitted Raman spectra of CrGO with different reducing agents and different reaction times.

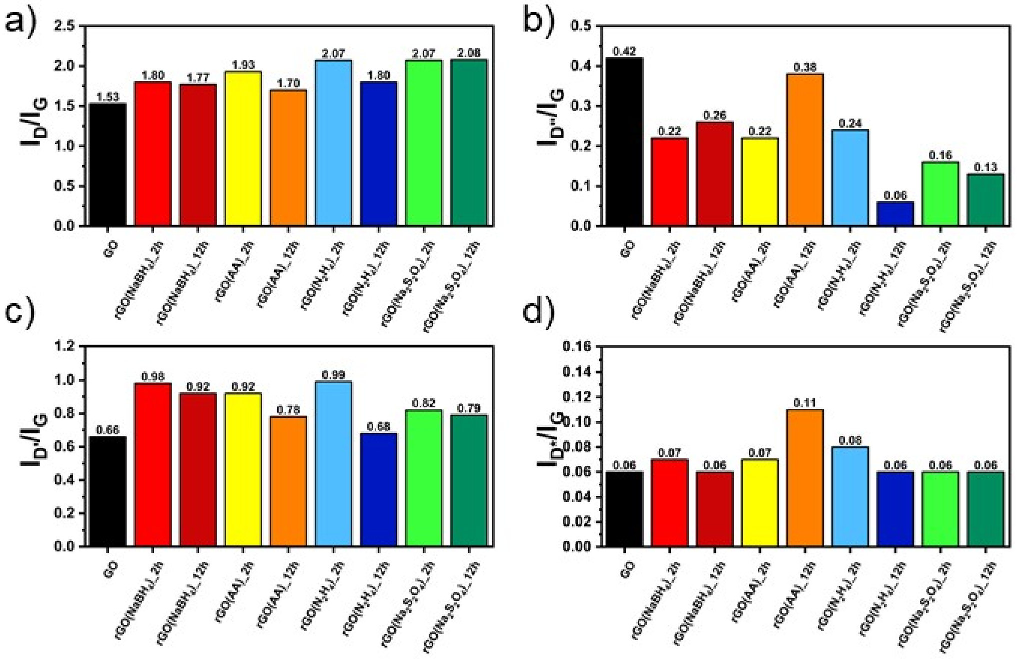


**Figure S13.** Ratio of the band intensities a) $I_D/I_G$ b) $I_{D''}/I_G$ c) $I_{D'}/I_G$ d) $I_{D*}/I_G$ of CrGO with different reducing agents and different reaction times.

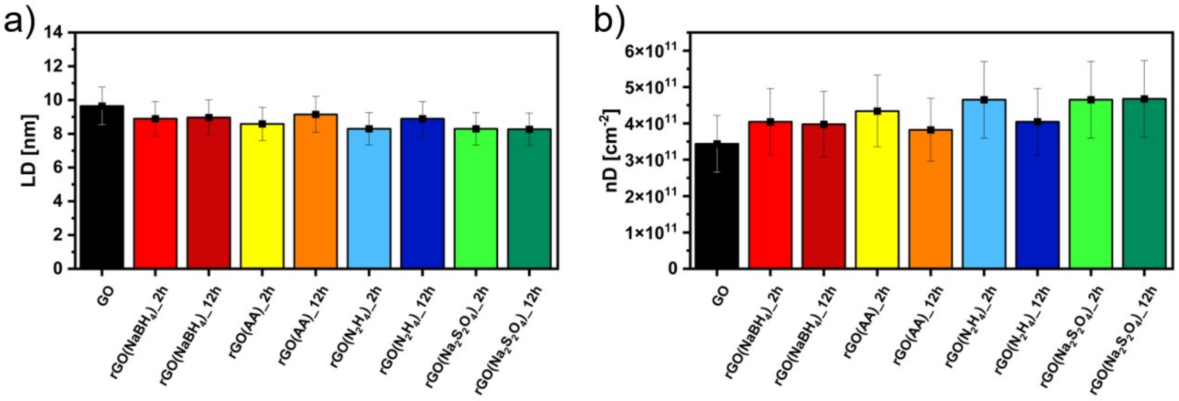


**Figure S14.** a) Average defect distance and b) defect density of CrGO with different reducing agents and different reaction times.

## Powder X-Ray Diffraction (PXRD)

### Calculation of the XRD parameters

From the XRD diffractograms the peak position has been calculated using the Bragg's law:

$$d_{(hkl)} = \frac{\lambda}{2\sin\theta} \tag{8}$$

Where $d_{(hkl)}$ is the calculated inter planar distance (Å), λ is the wavelength of the XRD source (Å), θ is the scattering angle (rad). The crystallite dimension has been derived from the Scherrer formula:

$$L_C = \frac{K\lambda}{\beta_a \cos\theta_a} \tag{9}$$

$$L_a = \frac{1.84\,\lambda}{\beta_b \cos\theta_b} \tag{10}$$

Where Lc is the crystallite thickness (Å), La is the crystallite size (Å), K is the shape factor equal to 0.89,[30] β is the FWHM of the (100) and (002) peaks and $\theta_a$ and $\theta_b$ are the corresponding scattering angles

**Table S5.** The XRD peak position, d-spacing, crystallite size ($L_a$), crystalline thickness ($L_c$) and average graphene layer number ($n_c$) calculated for the (002) and (100) planes for all measured samples.

| Sample | Reactant | Reaction time [h] | (0 0 2) position [°] | (1 0 0) position [°] | *d*-spacing [Å] | $L_a$ [Å] | $L_c$ [Å] | Number of layers $n_c$ |
|---|---|---|---|---|---|---|---|---|
| GO | - | - | 10.05 | - | 8.79±0.01 | 226.59±2.68 | 97.32±3.05 | 11.07 |
| rGO($NaBH_4$)_2h | $NaBH_4$ | 2 | 24.02 | 42.94 | 3.73±0.01 | 130.19±2.91 | 17.53±0.92 | 4.74 |
| rGO($NaBH_4$)_12h | | 12 | 23.82 | 943.08 | 3.70±0.01 | 117.93±1.59 | 14.75±0.49 | 3.95 |
| rGO(AA)_2h | AA | 2 | 23.55 | 43.01 | 3.77±0.01 | 104.49±1.88 | 13.81±0.67 | 3.66 |
| rGO(AA)_12h | | 12 | 24.24 | 42.85 | 3.67±0.01 | 92.28±1.46 | 15.24±0.70 | 4.16 |
| rGO($N_2H_4$)_2h | $N_2H_4$ | 2 | 23.08 | 42.97 | 3.85±0.01 | 93.30±2.99 | 12.52±0.77 | 3.25 |
| rGO($N_2H_4$)_12h | | 12 | 23.34 | 42.74 | 3.81±0.01 | 100.77±2.33 | 14.19±0.71 | 3.73 |
| rGO($Na_2S_2O_4$)_2h | $Na_2S_2O_4$ | 2 | 23.51 | 42.79 | 3.78±0.01 | 107.64±3.32 | 18.23±0.79 | 4.82 |
| rGO($Na_2S_2O_4$)_12h | | 12 | 23.77 | 42.85 | 3.74±0.01 | 99.66±1.71 | 17.95±0.69 | 4.80 |

**Scanning electron microscopy (SEM)**

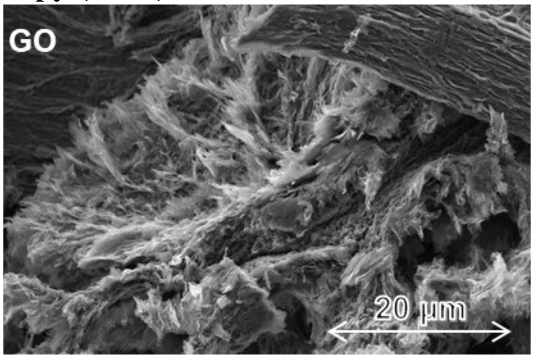


**Figure S15.** SEM image of GO.

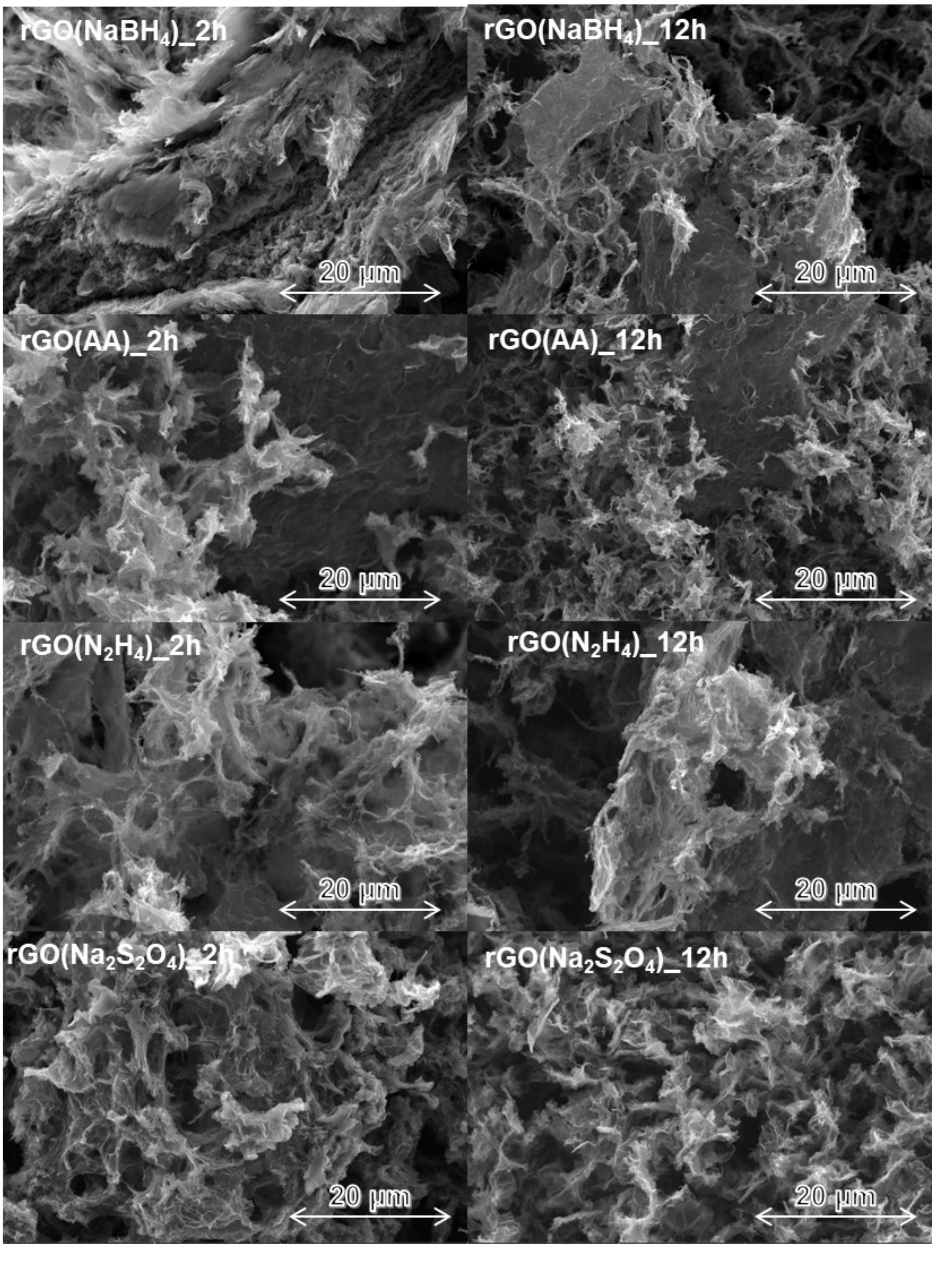

**Figure S16.** SEM images and CrGO with different reducing agents and different reaction times.

## Thermogravimetric analysis (TGA)

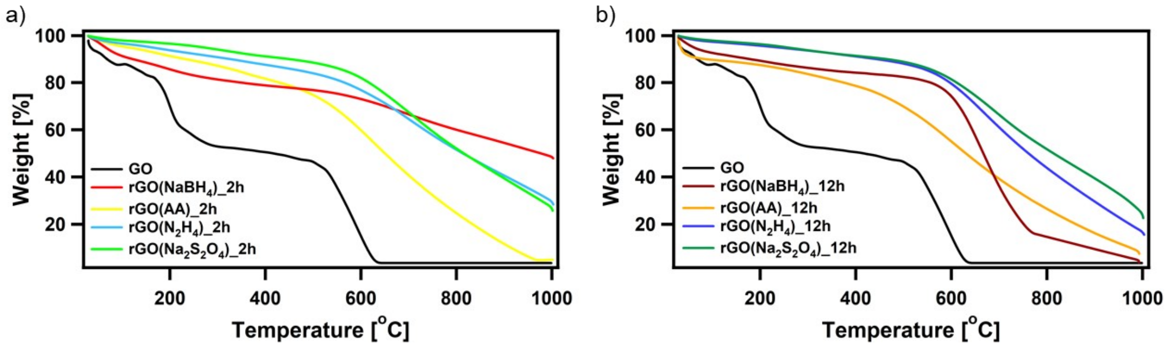


**Figure S17.** TGA diagram for the CrGO with different reducing agents at a) 2 and b) 12 hours of reaction time.

## BET characterization

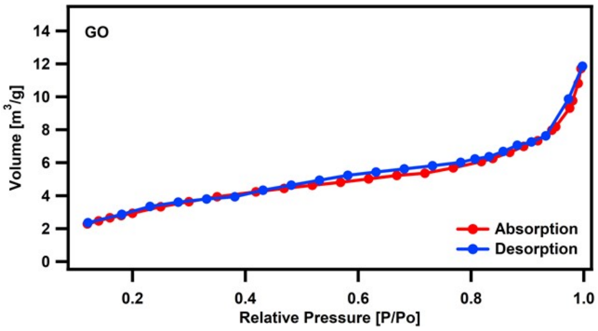


**Figure S18.** BET surface area for GO.

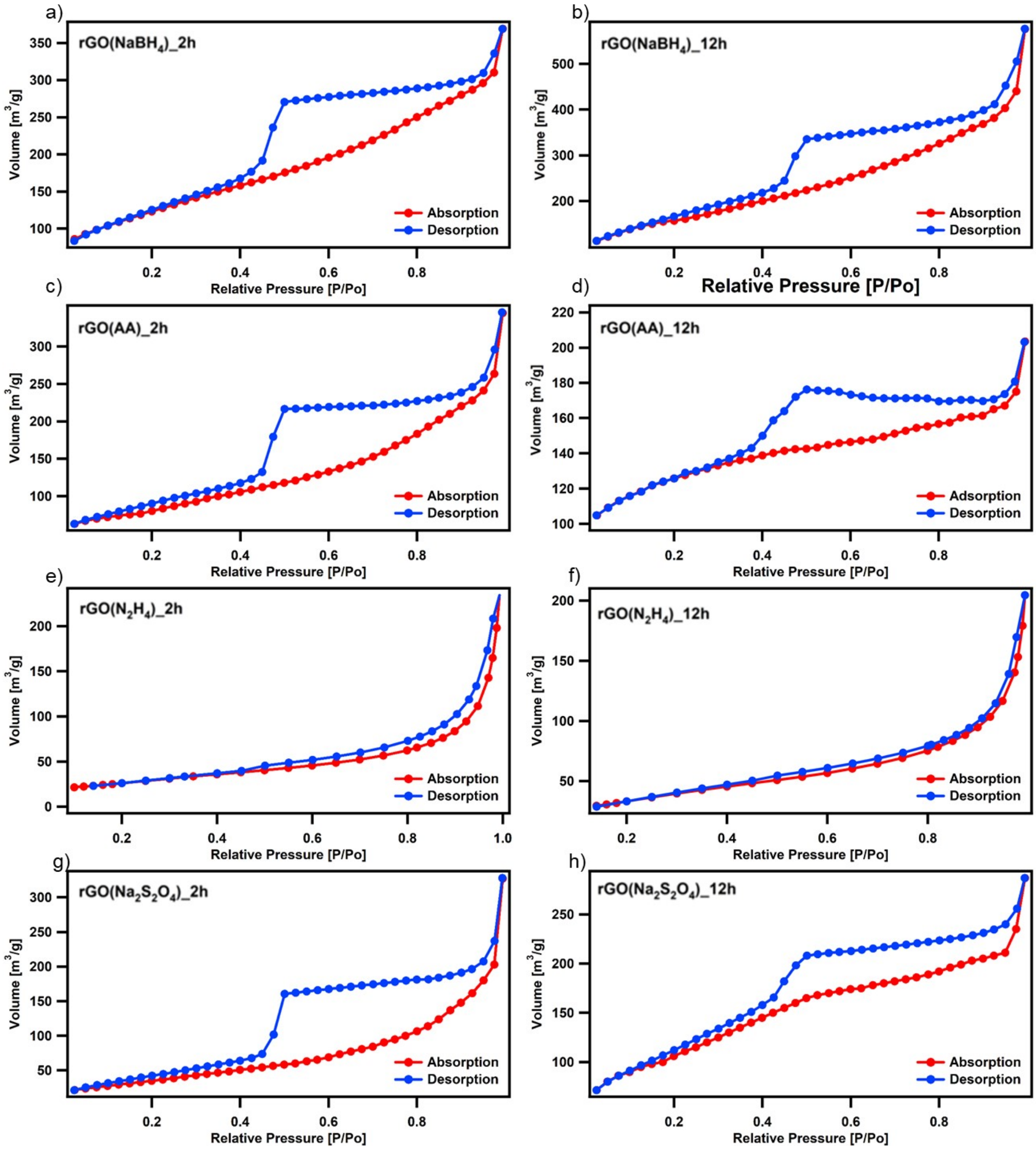


**Figure S19.** BET surface area for CrGO with different reducing agents and different reaction times.

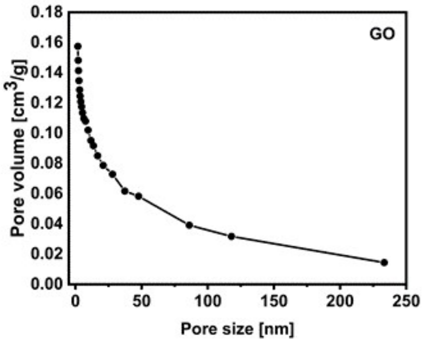


**Figure S20.** Pore size distribution of GO.

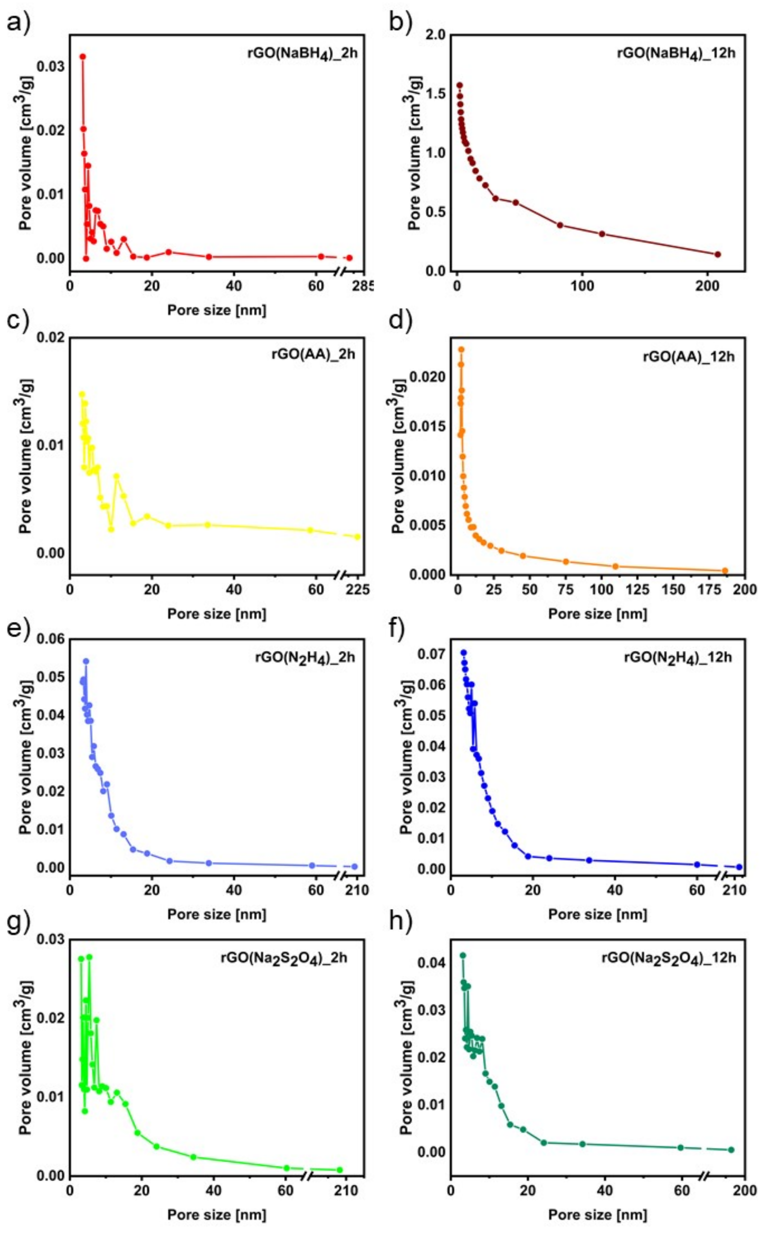


**Figure S21.** Pore size distribution of CrGO with different reducing agents and different reaction times.

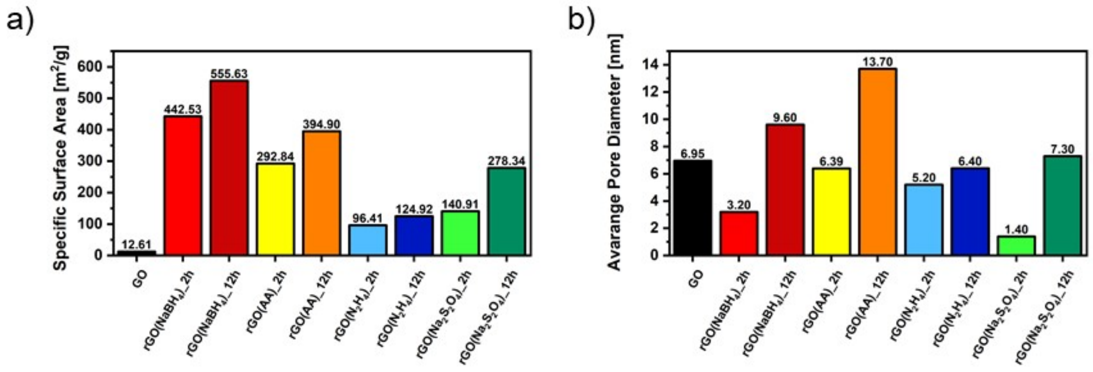


**Figure S22.** a) Specific surface area and b) average pore diameter obtained from BET isotherms.

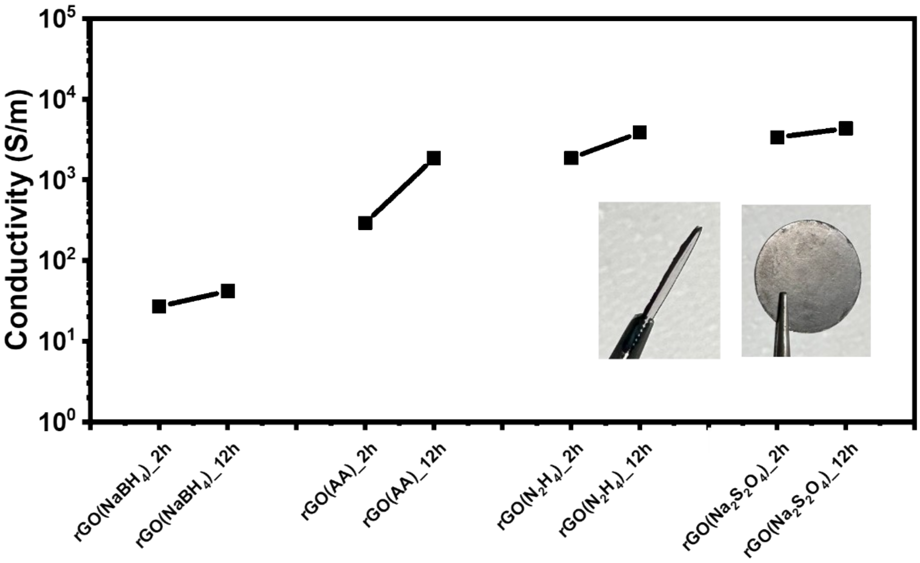


**Figure S23.** Film conductivity of CrGO with different reducing agents and different reaction times. Inset, lateral and frontal side of a representative pellet.

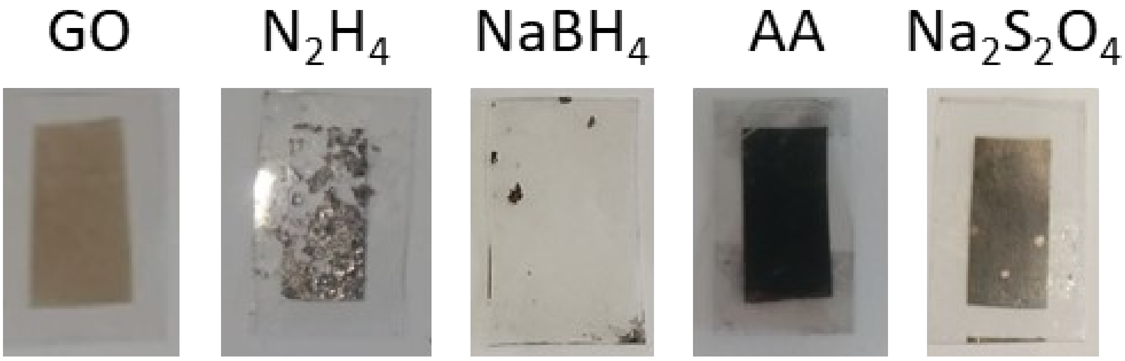


**Figure S24.** Optical images of the films of GO and CrGO with different reducing agents.

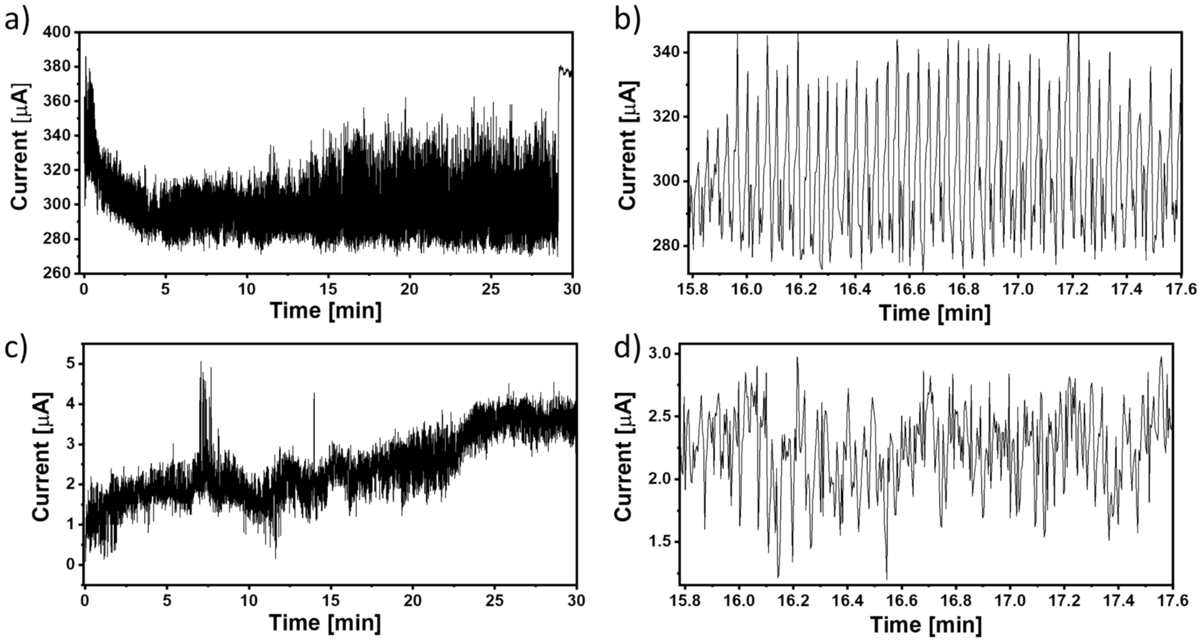


**Figure S25.** a, c) Resistance of the CrGO film obtained with a) $Na_2S_2O_4$ and c) AA, as a function of time during the application of 2000 bending cycles, b, d) magnification of the film resistance of CrGO obtained with b) $Na_2S_2O_4$ and d) AA, as a function of time.

## Section C. Electrochemical characterization

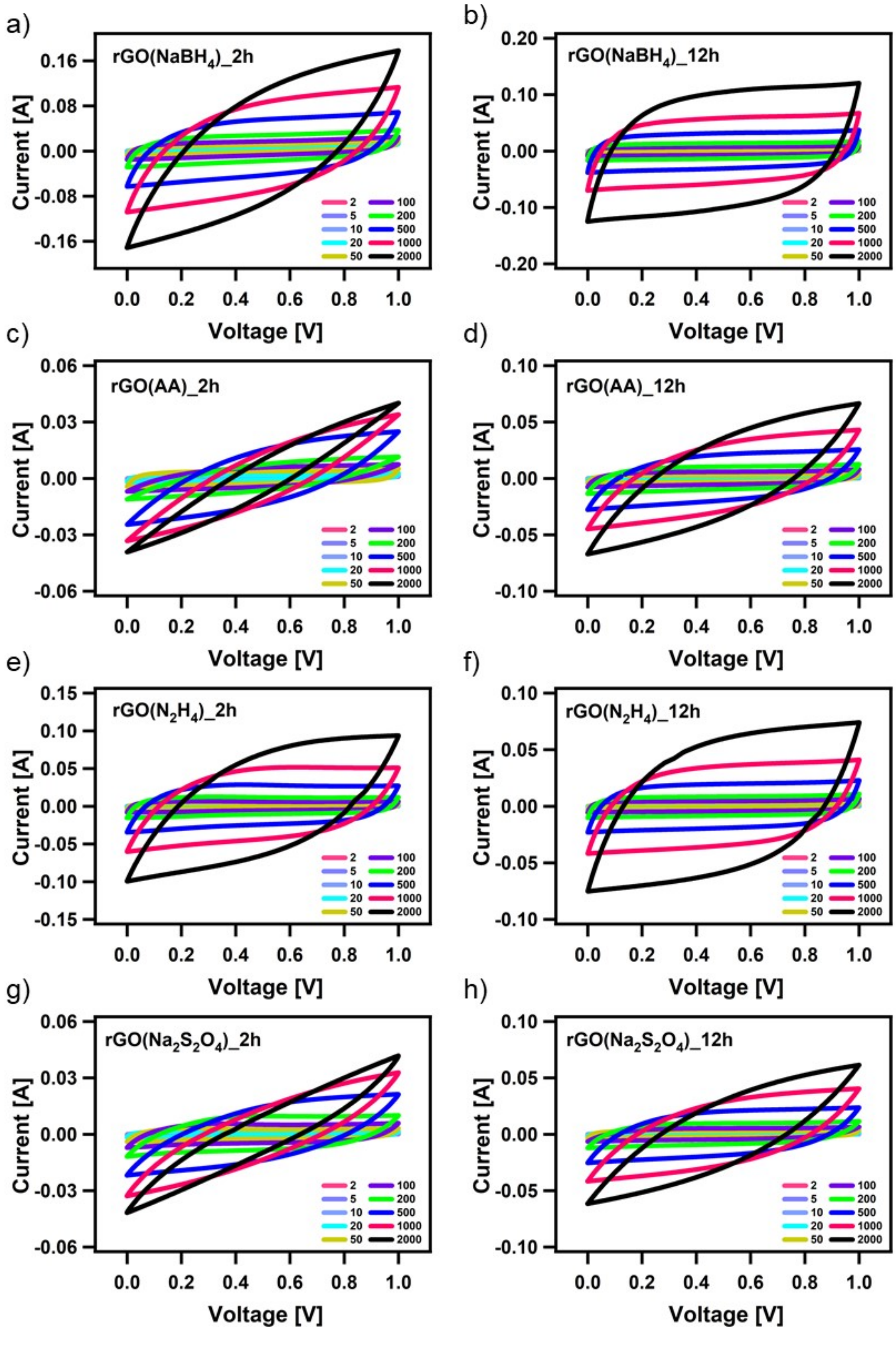


**Figure S26.** CV curves of CrGO with different reducing agents at different reaction times at different scan rates. The units of scan rate are mV/s.

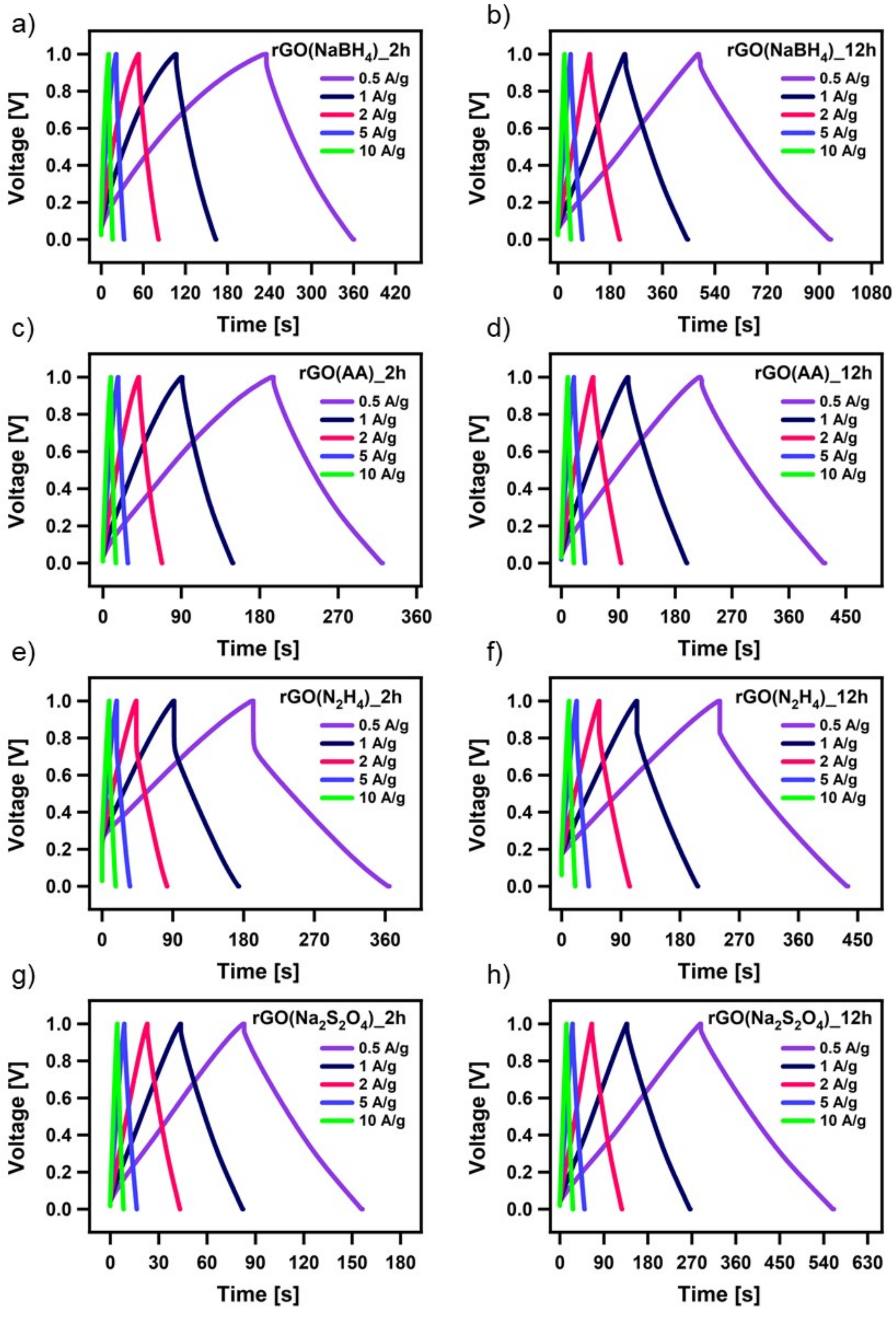


**Figure S27.** GCD curves of CrGO with different reducing agents and different reaction times at different scan rates.

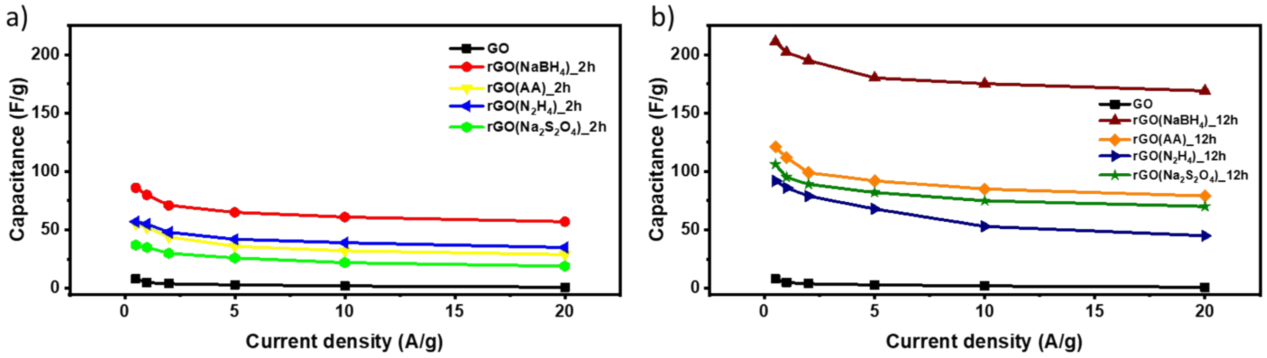


**Figure S28.** Comparison of specific capacitances versus current densities of CrGO with different reducing agents at a) 2 and b) 12 hours of reaction time.

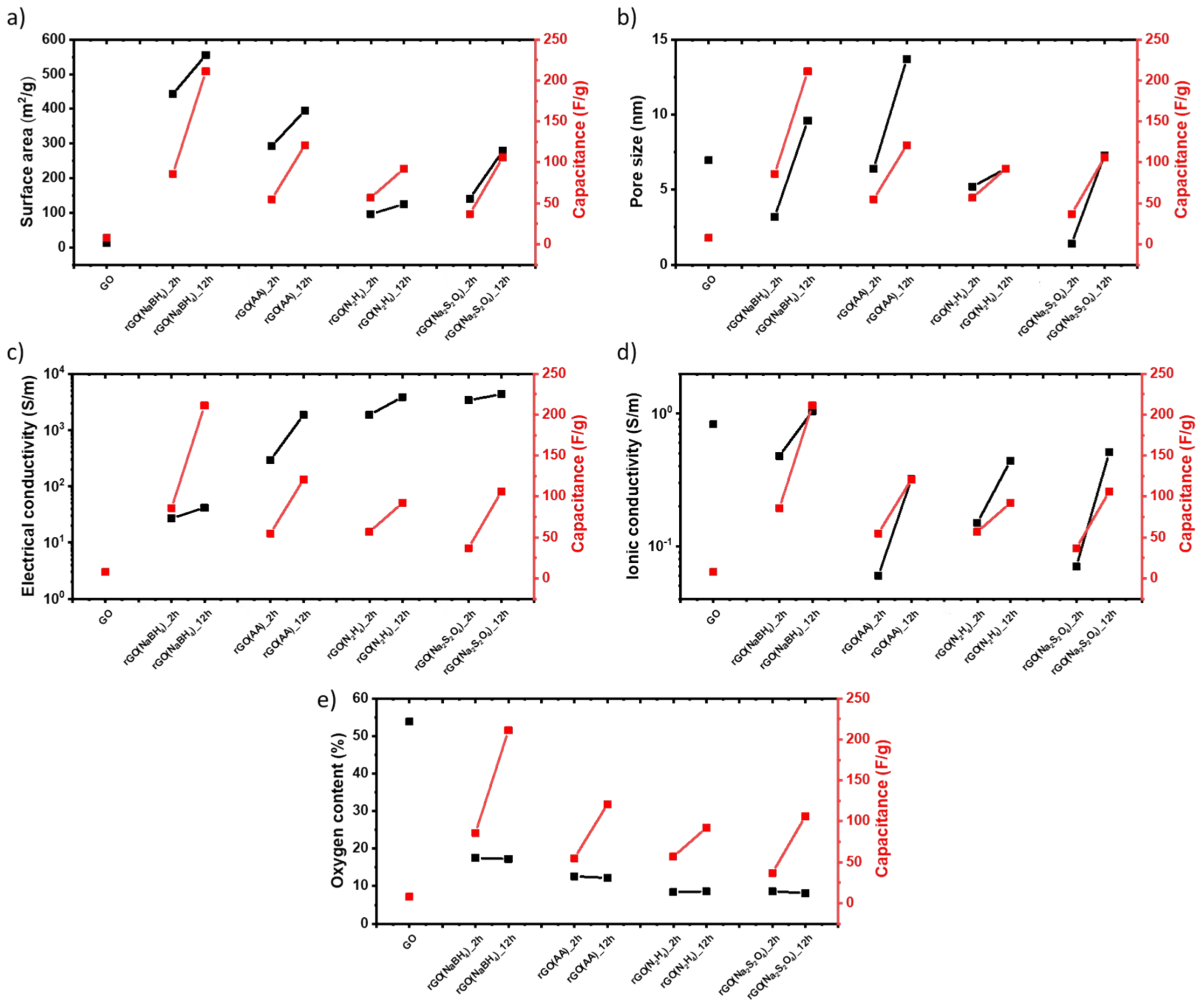


**Figure S29.** Specific capacitances at 0.5 A/g of CrGO with different reducing agents and reactions times compared with their corresponding (a) surface area, (b) pore size, (c) electrical conductivity (d) ionic conductivity and (e) oxygen content. Ionic conductivities were calculated by EIS.

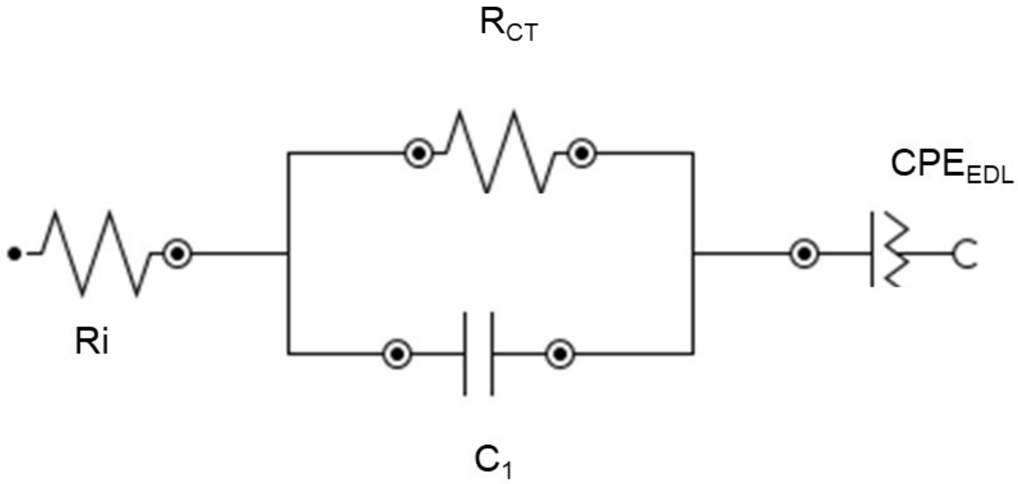


**Figure S30.** The equivalent electric circuit models used for fitting the Nyquist plots. Ri: the bulk resistance; Rct: charge transfer resistance; $C_1$: capacitance element; $CPE_{EDL}$: constant phase element representing the electrical double layer capacitance (EDLC).

**Table S6.** Fitting parameters obtained from the Nyquist plots.

| Sample | Ri (Ω) | $R_{CT}$ (Ω) | σ (S/m) |
|---|---|---|---|
| GO | 0.95 ± 0.04 | 12.33 ± 0.61 | 0.83 |
| rGO($NaBH_4$)_2h | 1.66 ± 0.02 | 1.45 ± 0.03 | 0.48 |
| rGO($NaBH_4$)_12h | 0.76 ± 0.01 | 2.21 ± 0.03 | 1.04 |
| rGO(AA)_2h | 13.31 ± 0.02 | 1.71 ± 0.03 | 0.06 |
| rGO(AA)_12h | 2.43 ± 0.02 | 2.09 ± 0.03 | 0.32 |
| rGO($N_2H_4$) _2h | 5.18 ± 0.02 | 0.63 ± 0.02 | 0.15 |
| rGO($N_2H_4$)_12h | 1.79 ± 0.02 | 3.99 ± 0.07 | 0.44 |
| rGO($Na_2S_2O_4$)_2h | 10.55 ± 0.03 | 3.20 ± 0.04 | 0.07 |
| rGO($Na_2S_2O_4$)_12h | 1.56 ± 0.01 | 0.70 ± 0.01 | 0.51 |

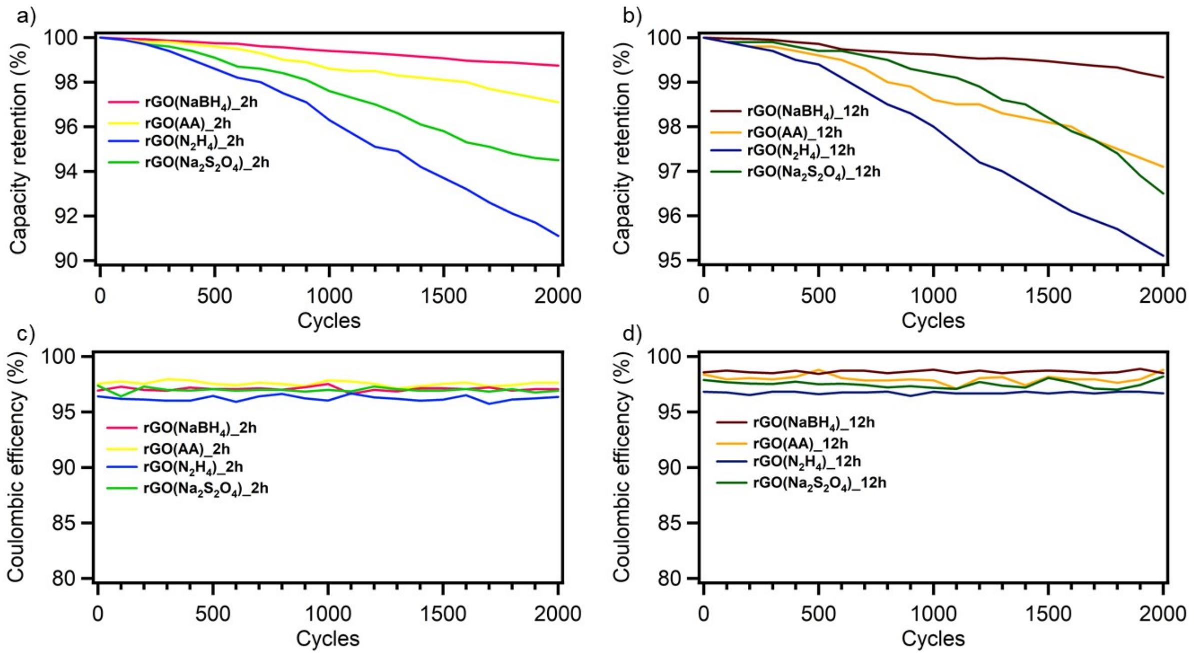


**Figure S31.** a), c) Cycling stability and b), d) coulombic efficiency of CrGO at the current density of 1 A/g.

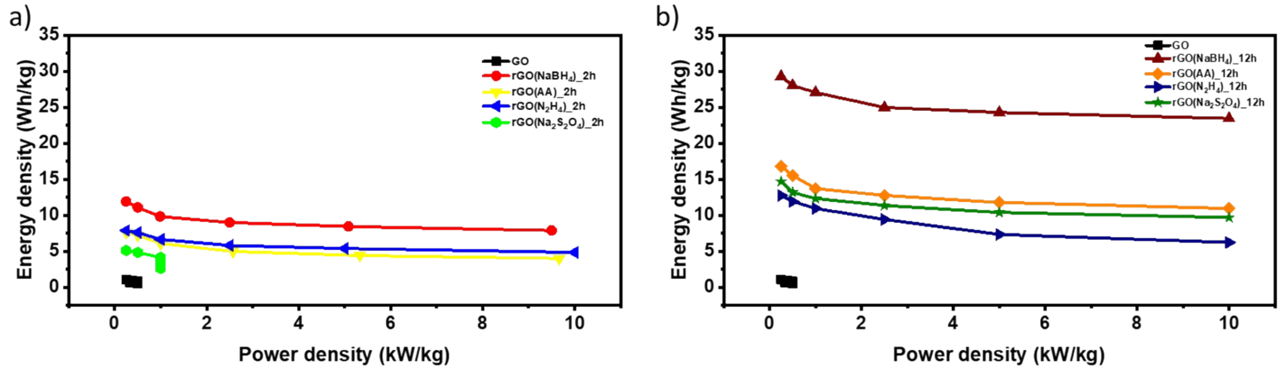


**Figure S32.** Ragone plot for CrGO using different reducing agents at a) 2 and b) 12 hours of reaction time.

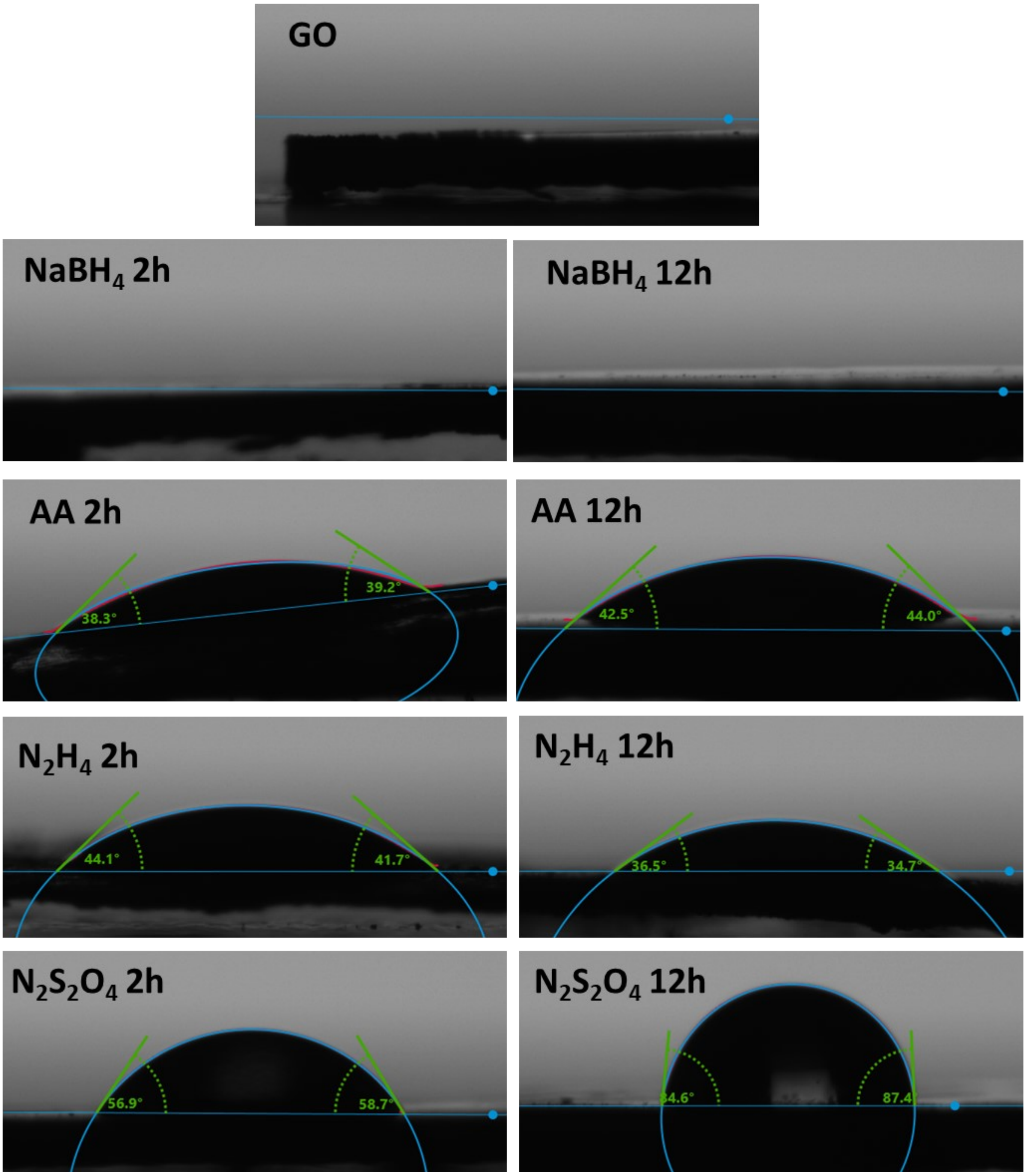


**Figure S33.** Optical images of the water sessile drop at different times on the CrGO-based films with the fitted contact angle represented.

## References


1. M. D. Stoller and R. S. Ruoff, *Energy Environ. Sci*, 2010, **3**, 1294-1301.
2. L. Fusco, M. Garrido, C. Martín, S. Sosa, C. Ponti, A. Centeno, B. Alonso, A. Zurutuza, E. Vázquez, A. Tubaro, M. Prato and M. Pelin, *Nanoscale*, 2020, **12**, 610-622.
3. N. Guo, Y. Cui, Y. Lin, S. Su, M. Li and X. Zhu, *Journal of Materials Science: Materials in Electronics*, 2022, **33**, 18294-18310.
4. Y.-M. Wang and C.-H. Zhang, *Nanomaterials*, 2022, **12**, 2989.
5. R. Li, L. Lai, S. Su, H. Dai, Y. Cui and X. Zhu, *Materials Today Communications*, 2020, **23**, 101128.
6. C. Bing, Y. Jiahao, L. Xiaoying, J. Qi, W. Guoping and J. Linghua, *Diamond and Related Materials*, 2021, **114**, 108305.
7. J. L. Zhang, H. J. Yang, G. X. Shen, P. Cheng, J. Y. Zhang and S. W. Guo, *Chem. Commun.*, 2010, **46**, 1112-1114.
8. X. Zhang, Z. Sui, B. Xu, S. Yue, Y. Luo, W. Zhan and B. Liu, *Journal of Materials Chemistry*, 2011, **21**, 6494-6497.
9. M. J. Fernández-Merino, L. Guardia, J. I. Paredes, S. Villar-Rodil, P. Solís-Fernández, A. Martínez-Alonso and J. M. D. Tascón, *The Journal of Physical Chemistry C*, 2010, **114**, 6426-6432.
10. C. Xu, X. Shi, A. Ji, L. Shi, C. Zhou and Y. Cui, *PLOS ONE*, 2015, **10**, e0144842.
11. R. Shadkam, M. Naderi, A. Ghazitabar, A. Asghari-Alamdari and S. Shateri, *Ceram. Int.*, 2020, **46**, 22197-22207.
12. W. Chartarrayawadee, S. E. Moulton, C. O. Too, B. C. Kim, R. Yepuri, T. Romeo and G. G. Wallace, *Journal of Applied Electrochemistry*, 2013, **43**, 865-877.
13. Y. Wang, Z. Shi, Y. Huang, Y. Ma, C. Wang, M. Chen and Y. Chen, *The Journal of Physical Chemistry C*, 2009, **113**, 13103-13107.
14. B. Rajagopalan and J. S. Chung, *Nanoscale Research Letters*, 2014, **9**, 535.
15. T. Zhou, F. Chen, K. Liu, H. Deng, Q. Zhang, J. Feng and Q. Fu, *Nanotechnology*, 2011, **22**, 045704.
16. N. Karim, S. Afroj, S. Tan, P. He, A. Fernando, C. Carr and K. S. Novoselov, *ACS Nano*, 2017, **11**, 12266-12275.
17. X. Zhou, J. Zhang, H. Wu, H. Yang, J. Zhang and S. Guo, *The Journal of Physical Chemistry C*, 2011, **115**, 11957-11961.
18. J. Han, L. L. Zhang, S. Lee, J. Oh, K.-S. Lee, J. R. Potts, J. Ji, X. Zhao, R. S. Ruoff and S. Park, *ACS Nano*, 2013, **7**, 19-26.
19. H.-J. Chu, C.-Y. Lee and N.-H. Tai, *Carbon*, 2014, **80**, 725-733.
20. Y. Chen, X. Zhang, D. Zhang, P. Yu and Y. Ma, *Carbon*, 2011, **49**, 573-580.
21. G. Shruthi, G. Baishali, V. Radhakrishna and P. Verma, *Graphene Technology*, 2020, **5**, 19-25.
22. H.-P. Cong, X.-C. Ren, P. Wang and S.-H. Yu, *Energy & Environmental Science*, 2013, **6**, 1185-1191.
23. X. Ji, Y. Song, J. Han, L. Ge, X. Zhao, C. Xu, Y. Wang, D. Wu and H. Qiu, *Journal of Colloid and Interface Science*, 2017, **497**, 317-324.
24. D. Chen, L. Li and L. Guo, *Nanotechnology*, 2011, **22**, 325601.
25. J. Li, G. Xiao, C. Chen, R. Li and D. Yan, *Journal of Materials Chemistry A*, 2013, **1**, 1481-1487.
26. C. K. Chua, A. Ambrosi and M. Pumera, *Journal of Materials Chemistry*, 2012, **22**, 11054-11061.
27. N. H. Kim, P. Khanra, T. Kuila, D. Jung and J. H. Lee, *Journal of Materials Chemistry A*, 2013, **1**, 11320-11328.
28. Y.-F. Li, Y.-Z. Liu, W.-K. Zhang, C.-Y. Guo and C.-M. Chen, *Materials Letters*, 2015, **157**, 273-276.
29. Z. Fan, K. Wang, T. Wei, J. Yan, L. Song and B. Shao, *Carbon*, 2010, **48**, 1686-1689.
30. D. J. Lim, N. A. Marks and M. R. Rowles, *Carbon*, 2020, **162**, 475-480.
